# Nitroxides: Chemistry, Antioxidant Properties, and Biomedical Applications

**DOI:** 10.3390/molecules30102159

**Published:** 2025-05-14

**Authors:** Krzysztof Gwozdzinski, Anna Pieniazek, Lukasz Gwozdzinski

**Affiliations:** 1 Department of Oncobiology and Epigenetics, Faculty of Biology and Environmental Protection, University of Lodz, 90-236 Lodz, Poland; krzysztof.gwozdzinski@biol.uni.lodz.pl (K.G.); anna.pieniazek@biol.uni.lodz.pl (A.P.); 2 Department of Pharmacology and Toxicology, Medical University of Lodz, 90-151 Lodz, Poland

**Keywords:** nitroxides, nitroxyl radicals, antioxidants, radioprotectors, proteins, EPR spectroscopy, MRI spectroscopy

## Abstract

Nitroxides are stable organic free radicals with a wide range of applications. They have found applications in chemistry, biochemistry, biophysics, molecular biology, and biomedicine as EPR/NMR imaging techniques. As spin labels and probes, they are used in electron paramagnetic resonance (EPR) spectroscopy in the study of proteins, lipids, nucleic acids, and enzymes, as well as for measuring oxygen concentration in cells and cellular organelles, as well as tissues and intracellular pH. Their unique redox properties have allowed them to be used as exogenous antioxidants. In this review, we have discussed the chemical properties of nitroxides and their antioxidant properties. Furthermore, we have considered their use as radioprotectors and protective agents in ischemia/reperfusion in vivo and in vitro. We also presented other applications of nitroxides in protecting cells and tissues from oxidative stress and in protein studies and discussed their use in EPR/MRI.

## 1. Introduction

Nitroxides (nitroxyls, nitroxyl radicals, and less often, aminoxyls) are stable free radicals with a wide range of applications. The presence of hydrophilic and hydrophobic substituents allows them to be directed to different regions in the cell. They are used in studies of proteins, lipids, nucleic acids, and enzymes, and also to measure oxygen concentration in cells and pH inside cell organelles as spin markers and probes in electron paramagnetic resonance spectroscopy (EPR). They have also been used in the spin-trapping method to convert short-lived radicals into paramagnetic compounds with greater stability, which can be studied at room temperature [1]. They have also found applications in polymer chemistry, nanoparticles, and as contrast agents in medicine in magnetic resonance imaging (MRI). Nitroxides, as stable organic radicals, have found numerous applications in organic synthesis due to their unique redox properties and reactivity. They are used in polymer synthesis and chemistry as stoichiometric and catalytic oxidants. Moreover, nitroxides serve as an efficient, safe, and environmentally friendly alternative to transition metal catalysts. In addition to the oxidation of alcohols, amines, and carbanions, as well as C-C bond formation reaction in both homogeneous and heterogeneous catalysis, nitroxides are also used as scavengers of carbon-center radicals. They also participate in nitroxide-mediated polymerization (NMP). In addition, they are used as catalysts in the oxidation reactions of primary and secondary alcohols to the corresponding aldehydes, ketones, and acids. However, in most reactions, the active oxidant is not the nitroxide but the oxoammonium cation/salt as its oxidized form [2]. Recently, Frémy’s salt (potassium nitrosodisulfonate), one of the first nitroxides, was used in the synthesis of a fixed gel network. In contrast to enzymes, whose diffusion could be limited by the size of the gel mesh, nitroxide can penetrate the network much better [3]. Electron paramagnetic spectroscopy (EPR or ESR) is an increasingly useful analytical tool for studying the structure of biomolecules, their dynamic behavior, and their interactions. Nitroxyl radicals are the most commonly used radical probes in EPR experiments, and many methods have been developed for their synthesis or incorporation into biomolecules using site-directed spin labeling. Recently, the most practical methods of nitroxide synthesis have been discussed, related to the possibility of tunable structures, modifications of their side chains in spin labeling, and their assembly in biomolecules [4]. Despite their initial limitations, nitroxides have been incorporated into macromolecular architectures, which is yet another application. The development of nitroxide-containing radical polymers has led to several remarkable breakthroughs in various fields, such as medical imaging (EPR, MRI) and energy storage applications [5]. The spin labeling technique in EPR spectroscopy pioneered by McConnell and colleagues (1965) is an increasingly useful method used to study the structure of macromolecules, and their dynamics or interactions. This method combined with directed spin labeling provides information on the structure and dynamics of proteins under conditions similar to their physiological environment. The information is based on the study of the local dynamics of a spin label, which is attached to the protein as a side chain of a selected amino acid. The analysis allows us to obtain reliable distance distributions between two or more labels, the same or different. The average value, width, and shape of the distance distributions, as well as their dependence on the protein state or interactions with physiological partners, provide insight into order-disorder transitions and the role of protein flexibility [6]. In addition, nitroxides are used as antioxidants, spin probes in oximetry, pH, or redox status in cells. They have been used as contrast agents in magnetic resonance imaging (MRI). Although initially overshadowed by the stronger paramagnetic metal ions, in their current form as polymer components they are also outdated and, importantly, less toxic. They are useful in vivo because they can reverse contrast enhancement after the administration of reducing agents [7].

Electron paramagnetic resonance is a noninvasive spectroscopic method that allows the monitoring of drug release processes in vitro and in vivo. In addition, spatial localization can be imaged using EPR-Imaging. In addition, new methods of in vivo spectroscopy and imaging have emerged, such as longitudinally detected EPR (LODEPR) and proton-electron double resonance imaging (PEDRI). Examples of EPR applications in the field of drug delivery allow for the measurement of microviscosity and micropolarity, in vitro and in vivo drug release mechanisms, monitoring of microacidity in biodegradable polymers, and characterization of colloidal drug carriers. Due to specific redox properties related to their oxidation and/or reduction, nitroxides have found application in protecting cells and tissues from oxidative stress in cells, tissues, organs, experimental animals, and even humans [8].

In this review, we discussed the chemical properties of nitroxides and their antioxidant properties. Furthermore, we considered their use as radioprotectors and protective agents in ischemia/reperfusion in vivo and in vitro. We also presented other applications of nitroxides in the protection of cells and tissues against oxidative stress and discussed their use in protein studies and EPR/MRI techniques.

## 2. Chemical Properties of Nitroxides

Nitroxides are stable organic radicals containing a nitroxide group N-O, which has an unpaired electron delocalized in a three-electron πN–O bond. This π bond is formed by the overlap of 2pz orbitals of nitrogen and oxygen atoms. The stability of nitroxyl radicals is related to the high delocalization energy of the strong three-electron bond πN–O (23–30 kcal mol^−1^) [9]. The nitroxyl group can be presented in the form of two basic mesomeric structures, which determine the stability of nitroxyl radicals:

>N-O^•^ → >N^+^-O^−^

**Figure 1:**
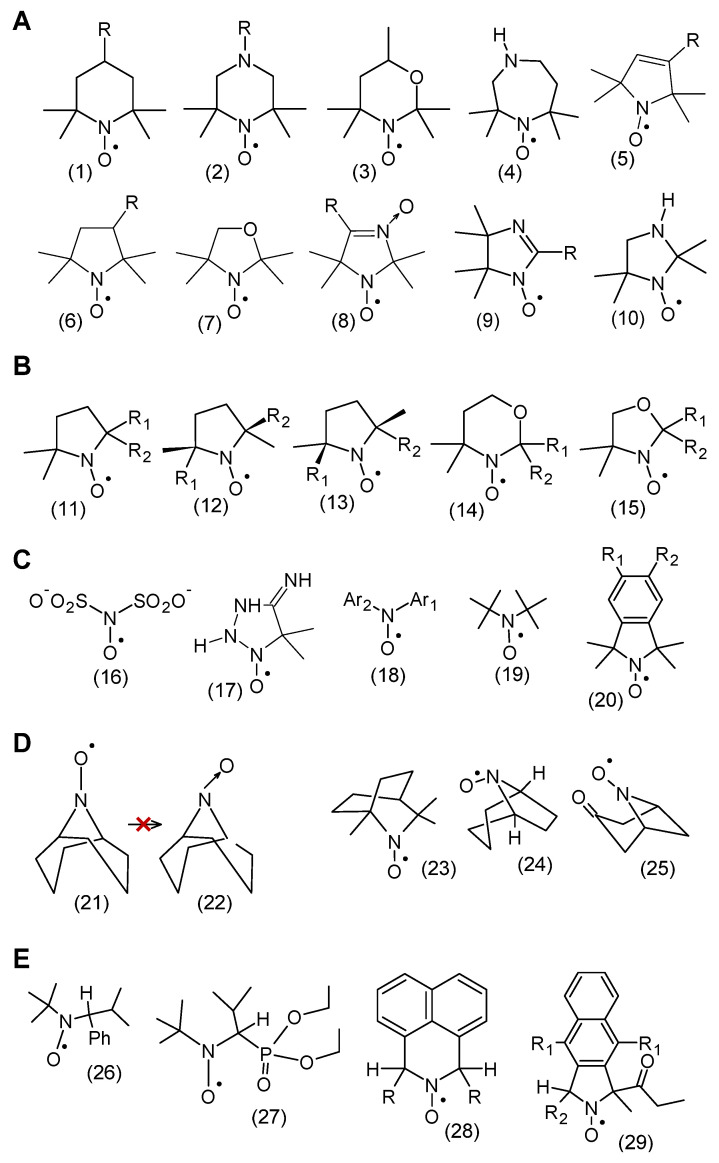
(**A**) Structures of various groups of heterocyclic nitroxides. Piperidine (**1**), piperazine (**2**) and oxazidine (**3**) derivatives and homopiperazine derivatives (1,4-diazepine) (**4**), as well as in nitroxides of five-membered derivatives of pyrroline (**5**), pyrrolidine (**6**), oxazolidine (**7**), imidazoline (**9**), imidazolidine (**10**). (**B**) Proxyl (**11**), azethoxyl (**12**, **13**), and oxazidin (**14**) and oxazolidine (**15**) nitroxides. (**C**) Fremy salt (**16**), porphyrexide (**17**), diarylnitroxide (**18**), di-tert-butyl nitroxide (**19**), isoindoline nitroxide. (**D**) Bredt’s rule, bicyclic nitroxide **21** and its nitron **22**. Various structures of bicyclic nitroxides include the following: trimethylisoquinuclidine *N*-oxyl (**23**), nortropane *N*-oxyl (**24**), 9-azabicyclo[3.3.1]nonane *N*-oxyl (**25**) [15]. (**E**) Aliphatic nitroxides having α-hydrogen atom, Tipno (**26**) and SG1 (**27**) as well as aromatic derivatives of naphthalene (**28**) and isoindoline (**29**).

The spin density between N and O atoms depends on such parameters as the pyramidalization of the nitrogen atom, resonance effects, and the polarity of the environment. For example, the first structure is dominant in a nonpolar environment, while the second structure is dominant in a polar environment [10]. In addition, the stability of nitroxides is related to the lack of hydrogen atoms at α-carbon atoms adjacent to the nitroxyl group, i.e., it is surrounded by quaternary carbon atoms (sp^3^ hybridization). This rule applies to many heterocyclic nitroxides, in which the nitroxyl group is surrounded by four methyl groups in nitroxides of six-membered piperidine and oxazidine derivatives, and seven-membered homopiperazine derivatives (1,4-diazepine), as well as in nitroxides of five-membered derivatives of pyrroline, pyrrolidine, oxazolidine, imidazoline, imidazolidine, and others (Figure 1) [11,12,13,14]. In practice, oxazolidine derivatives have various alkyl substituents in position 2.

A separate group of nitroxides are pyrrolidine derivatives having substituents in position 5,5, called Proxyl radicals [16]. Yet another group is represented by pyrrolidine derivatives having substituents in position 2,5, called azethoxyl radical, as well as 4,4,6-trimethyl-1,3-oxazidine-1-oxyl derivatives (Figure 1B) [17].

The first known nitroxyl radical (inorganic) was Frémy salt discovered in the second half of the nineteenth century and its synthesis was described by [18]. In turn, the first heterocyclic nitroxides, the so-called porphyroxides, were synthesized at the beginning of the twentieth century by Piloty and Schwerin (1901) [11]. The first aromatic nitroxyl radicals (di-phenynitroxide) were obtained by Wieland (Figure 1C) [19,20].

Nitroxides containing a hydrogen atom at the α-carbon atom are not stable and are easily disproportionated, forming the corresponding hydroxylamine and nitrone. However, there are exceptions to this rule, as many nitroxides are known to have hydrogen atoms at the alpha carbon atoms [15]. These include bicyclic and polycyclic radicals that do not undergo disproportionation reactions because this would require the formation of a carbon-nitrogen double bond with a bridgehead atom, which would be contrary to Bredt’s rule (Figure 1D).

In turn, steric hindrance, which prevents the formation of a nitrone structure, determines the stability of some aliphatic-aromatic radicals with hydrogen atoms at the alpha carbon atoms.

Other stable nitroxyl radicals are also known for having hydrogen atoms adjacent to α-carbon atoms, such as aliphatic Tipno and SG1 as well as aromatic derivatives of isoindole and naphthalene. The stability of compounds **1**, **2** having a-hydrogen atoms adjacent to the nitroxyl group is related to a combination of steric and stereoelectronic effects, which makes disproportionation kinetically impossible. In turn, the stability of naphthalene and isoindole derivatives was achieved by introducing two substituents R_1_, which lie in the plane of the aromatic ring (Figure 1E) [21].

**Figure 2:**
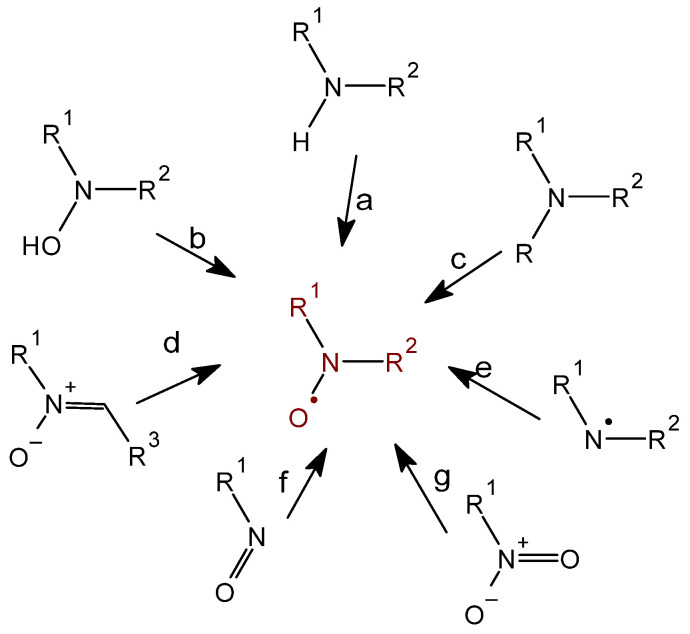
Different methods of nitroxide synthesis.

The most well-known preparative methods of nitroxides include oxidation of secondary amines and hydroxylamines (a, b) (Figure 2). These methods were used already in the late fifties of the last century to obtain piperidine, pyrroline, and pyrrolidine derivatives and were described in the work of Rozantsev and Ulrich [11]. Although much earlier methods of reducing nitroso- and nitro-aliphatic, aromatic and aliphatic-aromatic compounds to nitroxides were also known [11,22]. Nitroxyl radicals are also formed during the oxidation of tertiary amines (c) [12,23,24]. Nitroxides can also be obtained by oxidation of aminyl radicals with oxygen, but this is not a preparative method (Figure 2). The starting compounds for the synthesis of piperidine, pyrroline, and pyrrolidine derivatives were triacetonamine (2,2,6,6-tetramethylpiperidone-4) obtained by Heinz in 1874 or Tempone (4-oxo-2,2,6,6-tetramethylpiperidine-1-oxyl) (**53**).

The most commonly used oxidants in the preparation of nitroxides include lead oxide (II), silver oxide, manganese oxide (IV), potassium permanganate, potassium ferricyanide, nickel peroxide, sodium periodate, copper (II) compounds, hydrogen peroxide in the presence of sodium tungstate or phosphotungstic acid, peracids (m-chloroperbenzoic acid), hydroperoxides and organic peroxides, i.e., tert-butylhydroperoxide (t-BHP) and dibenzoyl peroxide, respectively [12]. Various nitroxides are obtained by carrying out reactions on the substituent of the starting heterocyclic amine and then oxidizing the amine group to the nitroxide. It is also possible to carry out reactions on the nitroxide by modifying only the substituent. It should be noted, however, that not all reactions can be carried out in two ways. Many reagents can react, for example, with the carbonyl group of triacetonamine and with the nitroxyl group, for example, hydrazine. The efficiency of this reaction, in which the Tempo (2,2,6,6-tetramethylpiperidine-1-oxyl) is formed, does not exceed 8%, and the remaining products are non-paramagnetic.

Another method of synthesis is the reaction of radicals with nitrones (N-oxides) known as spin trapping [10,25,26,27]. The most well-known spin traps include MNP) (2-methyl-2-nitrsopropan), PBN (N-tert-butyl-α-phenylnitrone), DMPO (5,5-dimethyl-1-pyrroline-N-oxide), and POBN (α-(4-pyridyl N-oxide)-N-tert-butylnitrone). Spin traps trapping short-lived radicals, such as hydroxyl radical, superoxide radical, methyl radical and others, transforming them into long-lived nitroxyl radicals, can be studied by EPR at room temperature.

Under certain conditions, nitroxides undergo reactions that lead to the disappearance of the EPR signal. These include disproportionation reactions in which hydroxylamine and oxoammonium salt are formed (e.g., reactions with strong acids). The substances oxidizing nitroxides include bromine, chlorine, and silver oxide. In turn, the reducing agents include ascorbic acid, hydrazine, phenylhydrazine, and organolithium compounds, and Grignard reagents, can reduce nitroxides to hydroxylamines. Nitroxides are also reduced to secondary amines, an example of which is their reduction using H_2_ in the presence of a palladium or Raney nickel catalyst, zinc or tin in hydrochloric acid, and alkali metal sulfides [11,12]. Reactions with free radicals formed as a result of ionizing radiation (γ and X) and UV radiation lead to oxoammonium salts, sometimes also to oxidation with the opening of the heterocyclic ring, with the formation of nitro derivatives [11].

## 3. Application of Nitroxides in Biology and Medicine

### 3.1. Immuno-Spin-Trapping

**Figure 3:**
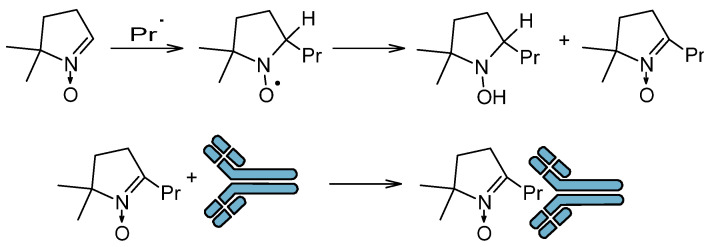
Protein radicals (Pr^•^) are formed as a result of the attack of alkyl, alkoxyl, and peroxide radicals on the protein molecule. Protein radicals are bound in situ by the DMPO spin trap, which forms a paramagnetic protein-radical adduct. Such an adduct disproportionates, forming a stable DMPO-protein nitrone, which can be identified using an anti-DMPO antibody and immunoenzymatic tests. In addition, the DMPO-protein nitrone can be isolated using immunoprecipitation from mixtures such as organelle homogenates, whole cells, or tissues and then studied using mass spectrometry, heterogeneous immunoassays, or molecular magnetic resonance imaging [28].

The spin trapping method has been extended to immuno-spin trap (IST), which involves the capture of a radical by a spin trap and then the use of antibodies to detect the nitrone adduct (Figure 3) [28]. This method does not use EPR spectroscopy. IST has found application in in vitro studies in which protein radicals are trapped. This method has been successfully used in models of drug-induced agranulocytosis, hepatotoxicity, cardiotoxicity, ischemia/reperfusion, and in models of neurological, metabolic, and immunological diseases [28]. This method allows the study of not only proteins but also cells, tissues, and experimental animals. Identification of altered proteins that are formed during the action of stressors on cells and tissues allows for specifying their function in the cellular response to stressors and pathogenesis. However, the obtained results cannot be treated uncritically, because usually high concentrations of the spin trap are used in the environment in which the radicals are formed. In turn, high concentrations of the trap can affect gene expression, metabolism, and physiology of the cell; therefore, interpretation of the obtained results requires great caution [28].

### 3.2. Antioxidant Properties of Nitroxides

**Figure 4:**
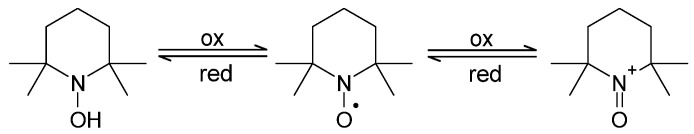
One-electron reactions of nitroxide reduction and oxidation. Reduction to hydroxylamine and oxidation to oxoammonium cation.

Numerous studies have shown that heterocyclic nitroxides have unique antioxidant properties that are related to their redox cycle. Nitroxides can undergo one-electron reduction in cells to the corresponding hydroxylamines or one-electron oxidation to oxoammonium salts (Figure 4). All three forms can coexist in cells and tissues. They participate not only in recombination reactions with other organic radicals but also in reactions of inorganic radicals, such as superoxide or hydroperoxide anion radicals and hydroxyl radicals. In addition, they exhibit pseudo-catalase properties in the inactivation of hydrogen peroxide. They also oxidize transition metal ions, inhibiting Fenton/Haber Weiss reactions. As non-toxic and non-immunogenic compounds, they are used in vivo as substances supporting endogenous antioxidant systems or modulating cellular redox systems. Additionally, nitroxides, by modifying the redox state of cells and tissues, can affect numerous metabolic processes. It has also been shown that nitroxides exhibit a protective effect against ionizing radiation and protect against oxidative damage to cells and tissues during ischemia/reperfusion. Nitroxides are also used as contrast agents in magnetic resonance imaging [7,29].

#### 3.2.1. Pseudodismutase Properties of Nitroxides

Nitroxides have full superoxide dismutase (SOD) mimetic properties in removing superoxide anion. In the first stage of the reaction, the nitroxide is oxidized by the superoxide anion to an oxoammonium ion:

R_1_R_2_N-O^•^ + O_2_^•−^ + 2H^+^ → R_1_R_2_N=O^+^ + H_2_O_2_

In the second stage, the oxoammonium ion is reduced by another superoxide anion molecule to nitroxide:

R_1_R_2_N=O^+^ + O_2_^•−^ → R_1_R_2_N-O^•^ + O_2_

Studies have shown that nitroxides easily penetrate cell membranes and can act as mimetics of superoxide dismutase, protecting cells and tissues from oxidative damage. Desferroxamine-Mn(III) chelate (DF-Mn) showed a similar effect. It was shown that neither SOD nor catalase (CAT) protected Chinese hamster V79 cell monolayers from the toxic effects of H_2_O_2_ or t-BHP. On the other hand, nitroxides and DF-Mn showed a protective role against these peroxides, which indicated the initiation of oxidative stress independent of O_2_^•−^ and other active forms derived from O_2_^•−^. The inhibition of damage by both metal-free and metal-based SOD mimetics can be attributed to the reaction of the SOD mimetic with a reduced transition metal to block the Fenton reaction and/or to the capture and detoxification of intracellular organic free radicals [30].

Other SOD mimetics based on metalloporphyrins and manganese-containing cyclic polyamines have also been developed. These substances can reduce other reactive oxygen species, such as peroxynitrite, carbonate anion radical (CO_3_^•−^) derived from peroxynitrite, superoxide radical, and, with lower efficiency, hydrogen peroxide [31]. Thus, SOD mimetics can reduce oxidative damage to cells and tissues. Nitroxide, like SOD, acts catalytically and is not consumed. Like endogenous SOD, nitroxide acts as a catalyst and the dismutation products of superoxide anion are hydrogen peroxide and oxygen and the nitroxide concentration is maintained at a constant level. The dismutation rate constants were lower at physiological pH by 2–3 orders of magnitude than SOD and ranged from 10^3^–10^6^ M^−1^ s^−1^ for different nitroxides [32,33]. Superoxide anion reacts very slowly (k < 10^3^ M^−1^ s^−1^) at physiological pH, but its protonated form (HO^•^_2_) reacts very rapidly (1.2 × 10^8^ M^−1^ s^−1^). In contrast, the reaction of O_2_^•−^ with the oxoammonium cation is much faster, limited only by the diffusion rate (k > 10^9^ M^−1^ s^−1^) [34]. In the absence of reducing agents, the oxoammonium cation has strong oxidizing properties and can oxidize many endogenous molecules and macromolecules, including DNA [35]. However, under reducing conditions, the oxoammonium cation can be reduced to hydroxylamine, which acts as an antioxidant by providing a hydrogen atom [36]. Such a reaction occurs in vivo in the case of thiols, alcohols, and NADH/NADPH [33,37,38]. On the other hand, NADH did not react directly with piperidine-derived nitroxides [37]. The oxidation of nitroxides by superoxide was evidenced by the inclusion of a two-electron reducing agent, such as NADH or NADPH, in the reaction. In their presence, the reduction of the nitroxide to the corresponding hydroxylamine by SOD was inhibited. At the same time, the oxidation of NADH or NADPH to NAD^+^ or NADP^+^ occurred [33,37]. The introduction of ethyl groups at the alpha position of the nitroxyl group in the piperidine ring prevented the reduction of the nitroxide in vitro by ascorbate or cytosolic extracts [39].

#### 3.2.2. Catalase Properties of Nitroxides

It has been shown that in the human body, 3% of hemoglobin is oxidized to methemoglobin (MetHb) daily, and a superoxide anion molecule is generated [40].

Hb(Fe^2+^O_2_) → Hb(Fe^3+^) + O_2_^•−^

In addition to their SOD-mimicking effect, nitroxides have been shown to induce the catalase properties of heme proteins in the removal of hydrogen peroxide. While nitroxides themselves do not decompose H_2_O_2_, they participate in the detoxification of hypervalent heme proteins, which include ferrylmyoglobin (MbFe^4+^=O) or the radical form of ferrylmyoglobin (^•^MbFe^4+^=O) formed during the oxidation of myoglobin (MbFe^2+^) and metmyoglobin (MetMbFe^2+^) with hydrogen peroxide, respectively. Hemoglobin behaves similarly.

Hb/Mb(Fe^2+^) + H_2_O_2_ → Hb/Mb(Fe^4+^=O^2−^) + H_2_O + O_2_

Hb/Mb(Fe^3+^) + H_2_O_2_ → Hb/Mb(Fe^4+^=O^2−^)^•+^ + H_2_O + O_2_

While the lifetime of the radical form of the ferryl form is measured in milisecunds, the lifetime of the oxoferryl form is expressed in minutes or hours. Tempol (4-hydroxy-2,2,6,6-tetramethylpiperidine-1-oxyl) led to a fourfold increase in catalase-like activity in the removal of H_2_O_2_ catalyzed by MetMbFe^3+^ with the evolution of molecular oxygen [41].

Hb/Mb(Fe^4+^=O)^•+^ + H_2_O_2_ → Hb/Mb(Fe^4+^=O) + O_2_^•−^ + 2H^+^

Hb/Mb(Fe^4+^=O) + H_2_O_2_ → Hb/Mb(Fe^3+^) + O_2_^•−^ + 2H^+^

The superoxide anion radical formed in the reaction undergoes spontaneous or SOD-catalyzed dismutation.

2H^+^ + O_2_^•−^ + O_2_^• −^ → H_2_O_2_ + O_2_

However, both ferryl species can oxidize the nitroxide to the oxoammonium ion [37,42].

Hb/Mb(Fe^4+^=O) + R_1_R_2_N-O^•^ → Hb/Mb(Fe^3+^) + R_1_R_2_N=O^+^

In turn, the oxoammonium ion oxidizes the hydrogen peroxide molecule to a superoxide anion radical:

R_1_R_2_N=O^+^ + H_2_O_2_ → R_1_R_2_N-O^•^ + O_2_^•−^ + _2_H^+^

Nitroxide, through the transformation between two oxidation states, i.e., the radical form and the oxoammonium cation, not only enhanced the catalase-like activity of MbFe^3+^ in the decomposition of H_2_O_2_ but also protected the macromolecules against oxidation by the reactive ferryl forms of Hb and Mb. In the presence of NADH/NADPH, the oxoammonium cation is reduced to an amine [41]:

R_1_R_2_ N=O^+^ + NAD(P)H → R_1_R_2_ N-H + NAD(P)^+^

#### 3.2.3. Nitroxides as Inhibitors of Haber–Weiss and Fenton-like Reactions

In the Fenton reaction, the reducing agent of hydrogen peroxide is the iron ion Fe^2+^:

Fe^2+^ + H_2_O_2_ → Fe^3+^ + HO^•^ + HO^−^

In the case of Fenton-like reactions, the reducing agents are transition metal ions in lower oxidation states, such as Cu, Ce, Ti, Mn, Ni, Fe, Cu, Co, Mn, Ce, Ag, Cr, Ru, W, Mo, V, and Ti. In the Fenton-like reaction, factors such as pH, ligands, multivalent metal structure, electron transfer, presence of oxygen, and presence of reducing agents are important [43]. In the Haber–Weiss reaction, the reducing agent is the superoxide anion radical:

O_2_^•−^ + H_2_O_2_ → O_2_ + HO^•^ + HO^−^

The Haber–Weiss reaction is kinetically slow, but in the presence of transition metal ions, this reaction becomes fast. Additionally, its rate is influenced by the factors mentioned above in the case of Fenton-like reactions. Nitroxides can prevent the generation of hydroxyl radicals in Fenton/Haber–Weiss reactions by oxidizing the metal ion catalyzing the reaction and/or by binding the OH radical [32,44,45]:

R_1_R_2_N-O + HO^•^ + 2H^+^ + Fe^2+^ → R_1_R_2_N-OH + Fe^3+^ + H_2_O

R_1_R_2_N-O + HO^•^ → R_1_R_2_N-OOH → R_1_R_2_N=O^+^ + OH^−^

#### 3.2.4. Reactions of Nitroxides with Radicals

Nitroxides have a protective effect by reacting with such forms of oxygen as singlet oxygen, peroxide radicals, and nitrogen dioxide [46,47] and a strong oxidizing and nitrating agent, peroxynitrite [48,49,50]. As stable radicals, nitroxides undergo recombination reactions with other free radicals, such as hydroxyl (HO^•^), alkyl (R^•^), and peroxide (ROO^•^) radicals but do not react with alkoxyl radicals (RO^•^) [51,52,53,54]. Tempo, on the other hand, reacted with alkyl radicals at a diffusion-limited rate (rate constant 1 − 3 × 10^10^ M^−1^ s^−1^) [52]. Recently, it was shown that the rate constants of the interaction of piperidine nitroxides with ROO^•^ radicals depend on the substituent at position 4 − 5.1 × 10^6^ M^−1^ s^−1^, Tempo as 1.1 × 10^6^ M^−1^ s^−1^ for Tempol and 5.4 × 10^5^ M^−1^ s^−1^ for Tempamine (4-amino-2,2,6,6-tetramethylpiperidine-1-oxyl) to 5.6 × 10^4^ M^−1^ s^−1^ for Tempon (4-oxo-2,2,6,6-tetamethylpiperidine-1-oxyl) [55]. Tempol was involved in the scavenging reaction of globin radicals. It acted as a catalyst, as the resulting oxoammonium cation generated during the reaction initiated secondary reactions that led to the free carbonyl at the N-terminal Gly residue of the protein. Tempol has also been shown to be useful for identifying radical formation sites on hemoproteins [56].

#### 3.2.5. Reactions of Nitroxides with Singlet Oxygen

The reaction of 2,2,6,6-tetramethyl-4-piperidinol with singlet oxygen was studied by EPR spectroscopy. The reaction rate constant of the amine was 8 × 10^5^ M^−1^ s^−1^ in ethanol and 4 × 10^7^ M^−1^ s^−1^ in phosphate buffer (pH 8) [57]. Singlet oxygen was shown to oxidize 2,2,6,6-tetramethyl-4-piperidone to nitroxide, but the yield was not very high. The addition of ascorbate, Fe^2+^, and desferrioxamine led to an increase in nitroxide production.

RR’N-H + ^1^O_2_ → RR’N-OOH → RR’N-O^•^ + ^•^OH

Homolytic cleavage of the O-O bond of N-hydroperoxide as an intermediate product is a slow process, which may be responsible for the relatively low production of nitroxide. The addition of electron “donors” to the reaction system increased the rate of hydroperoxide decomposition. The highest efficiency was observed in the presence of desferrioxamine. Interestingly, O_2_^•−^/HO_2_^•^ and ^•^OH radicals formed in a phosphate-buffer solution (pH 7.4) did not oxidize 2,2,6,6 tetramethyl-4-piperidone to nitroxide [58].

#### 3.2.6. Reactions of Nitroxides with Peroxynitrite

Nitroxides, or rather their oxidized forms (oxoammonium cation), are scavengers of peroxynitrite (k = 6 × 10^6^ M^−1^ s^−1^ for Tempo pH 5) [59]. The reduction potential for the RNO^+^/RNO^•^ pair varies in the range of 770–1000 mV relative to NHE for the RNO^+^/RNO^•^ pair), ring size, ring substituents, and charge [60]. In addition, nitroxides react with peroxynitrite products, i.e., carbonate anion radical (CO_3_^•−^) and nitrogen dioxide (NO^•^_2_) with a rate constant higher than 10^8^ M^−1^ s^−1^ at physiological pH [60]. Since nitrogen dioxide is a strong nitrating agent, its removal prevents the nitration of tyrosine to 3-nitrotyrosine [60]. Oxoammonium cation (Tempo) also reacts with nitric oxide (NO^•^) with a rate constant of 9.8 × 10^3^ M^−1^ s^−1^ [61]. Nitroxides also react with protein radicals in which the unpaired electron is located on tyrosine residues, as well as with phenoxy radicals, and those derived from tryptophan (carbon centers) and peroxide residues [61]. For example, Tempo reacts with peroxide radicals with a rate constant of 2 × 10^7^ to 10^8^ M^−1^ s^−1^, which depends on the type of radical [51]. The rate constant of the reaction with thiyl radicals is also high (5 − 7 × 10^8^ M^−1^ s^−1^) [62]. Nitroxides have been shown to prevent the modification of human serum albumin (HSA) by peroxynitrite and SIN-1, a peroxynitrite generator (morpholinosydnonimine, hydrochloride). Nitroxides protected the protein to a greater extent against nitration than against oxidation. Hydrophilic nitroxides protected MCF-7 cells against nitration by SIN-1 to a greater extent than hydrophobic ones [63].

#### 3.2.7. Reactions of Nitroxides with Hypochlorous Acid

Reactions of nitroxides with hypochlorous acid (HClO) as a result of activation of phagocytic cells, mainly neutrophils, produce many reactive oxygen species, including hypochlorous acid. Activation of neutrophils by various factors, including bacteria, is involved in the progression of many human inflammatory diseases. HClO is formed as a result of oxidation of chlorides by hydrogen peroxide in the presence of myeloperoxidase (MPO). In the first stage of the reaction, Compound I (Por(Fe^4+^=O)^•+^) is formed:

Por(Fe^3+^) + H_2_O_2_ → Por(Fe^4+^=O)^•+^ + 2H^+^ + O_2_^•−^

In the second stage, chlorides are oxidized:

It was shown that nitroxides strongly inhibited the production of HOCl dependent on MPO. The inhibition mechanism was associated with the single-electron oxidation of nitroxides by the form I of MPO and the accumulation of Compound II.

Por(Fe^4+^=O)^•+^ + R_1_R_2_N-O^•^ → Por(Fe^4+^=O) + R_1_R_2_N=O^+^

Since the reduction of compound two to the parent enzyme form (MPO(Fe^3+^) by nitroxide is slow, it inhibits HClO production [64].

Por(Fe^4+^=O) + R_1_R_2_N-O^•^ → Por(Fe^3+^) + R_1_R_2_N=O^+^

Thus, nitroxides are substrates for Compounds I and II. Some of the nitroxides led to heme destruction. The inhibition of neutrophil HOCl production by nitroxides was antagonized by neutrophil-derived superoxide, which was attributed to the superoxide-mediated reduction of Compound II. This effect was marginal for 4-amino-Tempo (**55**), probably due to the effective superoxide dismutase mimetic activity of this nitroxide. Overall, these data indicate that nitroxides have significant potential as therapeutic agents in inhibiting MPO-mediated damage in inflammatory diseases. Furthermore, nitroxides can limit protein nitration catalyzed by the MPO-H_2_O_2_- NO^•^_2_ complex—which was associated with the reduction of nitrogen dioxide to nitrite [65].

R_1_R_2_N-O^•^ + NO_2_^•^ → R_1_R_2_N=O^+^ + NO_2_^−^

### 3.3. Radioprotective Effect of Nitroxides

Radiation can be considered as the emission of waves or particles, which are classified as ionizing or non-ionizing radiation. Ionizing radiation has sufficient energy to ionize atoms or molecules. Ionizing radiation is high-energy electromagnetic radiation, which includes gamma rays and X-rays, but also radiation in the form of high-energy electrons, neutrons, and protons. Gamma radiation is sourced from radioactive elements (e.g., ^60^Co), and X-ray tubes from X-rays. On the other hand, non-ionizing radiation has lower energy and does not cause ionization of atoms and molecules. Ionizing radiation and radioactive substances are natural and permanent components of our environment. They are also used in industry, agriculture, medicine, and various research. Ionizing radiation is used in radiotherapy, radiology, and nuclear medicine, but it is dangerous to living organisms. High water content in cells and tissues promotes water radiolysis initiated by ionizing radiation.

H_2_O → H^•^ + ^•^OH + H^+^ + e_aq_

This reaction produces short-lived chemical entities (10^−9^–10^−10^ s), such as hydroxyl radicals, active hydrogen atoms, and hydrated electrons (e_aq_). Hydrated electrons react with ionized water molecules to form further hydroxyl radicals [66]. ^•^OH radicals, and hydrogen atoms can damage life-important molecules and macromolecules (LIMs), such as proteins, nucleic acids, lipids, sugars, and others. It is also possible to break chemical bonds in molecules and generate secondary electrons. Among many endogenous radioprotectors, e.g., thiols, glucose, and others, radioprotective action is also exhibited by other substances, such as nitroxides, which can regenerate damaged macromolecules:

R_1_R_2_N-OH + LIM^•^ + H^+^→ R_1_R_2_N=O^•^ + LIMH

Since in cells and tissues, some nitroxides are reduced by endogenous reductants to hydroxylamines, they can also scavenge radicals participating in electron transfer and regenerate radicals by donating a hydrogen atom, repairing the damaged molecule/macromolecule:

R_1_R_2_N-OH + LIM^•^ → R_1_R_2_N-O^•^ + LIMH

γ well-defined radiation is a convenient factor initiating oxidative damage. In addition, it provides known radicals and allows for the understanding of the mechanisms of macromolecule damage. γ radiation was used to damage small unilamellar vesicles made of egg phosphatidylcholine, which contains about 10% polyunsaturated fatty acids (PUFA). The degree of PUFA degradation was monitored using gas chromatography. Nitroxides, Tempo, and Tempol protected fatty acids from oxidative damage in a concentration-dependent manner. Interestingly, despite the large difference in hydrophobicity (Tempo ≫ Tempol), both nitroxides provided full protection against radiation [67]. Ionizing radiation is often used in cancer therapy, which is toxic not only to mutant cells but also to normal cells. The harmful effects of radiation on normal cells result in inflammation of the mucous membranes (mucositis), dry oral mucosa (xerostomia), tissue fibrosis, and induction of carcinogenesis. Often, lower doses of radiation are used for these reasons, but this reduces the effectiveness of the therapy. This is the reason for the growing interest in finding new, effective radioprotectors. Radioprotectors are substances (drugs) that are introduced into the body before the application of radiation, protect normal (non-cancerous) cells from damage caused by radiotherapy, and reduce and alleviate the effects of radiation. Radioprotectors include the FDA-approved, natural substance amifostine and the protein palifermin. Amifostine is a cytoprotectant that protects the kidneys, bone marrow, and heart in chemotherapy Most radioprotectors are compounds containing thiol groups, such as cysteine, cysteamine, cystamine, 2-(2-Aminoethyl)isothiourea, which undergo hydrolysis exposing the -SH group, and 2-mercaptoethylguanidine. Nitroxides and oxoammonium salts have been shown to have radioprotective activity in vitro and in vivo [68]. Furthermore, nitroxides are not immunogenic and mutagenic and do not exhibit toxicity to the human body. They protect healthy cells but do not protect cancer cells from ionizing radiation [69,70,71].

**Figure 5:**
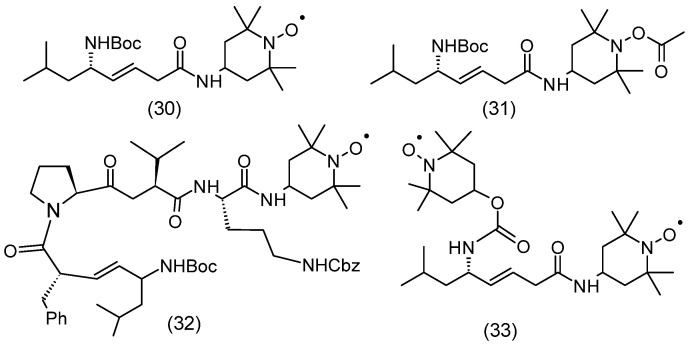
Structure of JP4-039 (**30**) and its derivatives (TK649-030 (**31**), XJB5-131 (**32**) hemigramicidin S (hemiGS)-nitroxide and JRS527.084 biradical (**33**) radiation mitigators localizing to mitochondria, obtained by combining the appropriate compounds with 4-amino-Tempo.

Esophagitis was a consequence of the use of radiotherapy in the destruction of non-small cell lung cancer and esophageal cancer. Esophagitis after radiotherapy limits the possibilities of dose escalation due to dehydration, esophageal ulceration, and the need for treatment breaks. JP4-039 (**30**) nitroxide administered intraesophageally was also shown to be effective in ionizing radiation-induced esophagitis (Figure 5). In general, this nitroxide promoted the recovery of endogenous esophageal progenitor cells [72]. Gramicidin S is a cyclodecapeptide, composed of two identical pentapeptides. It has antimicrobial activity. It is used only topically because of its hemolytic side effects, but it may be a promising therapeutic compound. The mitochondrial-targeting conjugate of hemigramicidin S (formula) with nitroxide (hemiGS-nitroxide) XJB-5-131 (**32**) and JP4–039 (**30**) is nontoxic over a wide range of doses and exhibits strong attenuating properties in response to radiation (Figure 5). The results of radiation attenuation in the hematopoietic syndrome of the GS-nitroxide derivative XJB-5-131, which showed more effective mitochondrial localization, were compared with two analogs of JP4-039, the mitochondrial-localizing biradicals JRS527.084 (**33**) and TK649.030 (**31**) (Figure 5). Mice given JP4-039 had the highest survival rate after 35 days (33%) compared to other groups treated at a dose of 9.5 Gy. The obtained results showed that JP4-039 exhibited the strongest radiation-mitigating properties [73]. During the action of ionizing radiation, in addition to many reactive oxygen species, superoxide and nitric oxide are also formed, which as a result of the reaction create a strong oxidant and nitrating agent, peroxynitrite. These factors oxidize cardiolipin (CL) in mitochondria, which causes the release of cytochrome c and activation of caspase, which consequently triggers the process of apoptosis. The introduction of Tempol enhanced cytoprotection against oxidative stress initiated by radiation. Nitroxide not only inactivates free radicals but also acts as a SOD imitator by capturing superoxide, which are precursors of other reactive oxygen species (ROS). Similar properties were demonstrated by hydroxylamine derived from nitroxide [74].

Salivary glands are among the glands that are very sensitive to radiotherapy, which causes xerostomia. Similarly to the previous experiment, the effect of Tempol (**53**) on radioprotection was studied in C3H mice, specifically on their salivary glands. The compound was administered 10 min before irradiation with different doses of radiation. It turned out that Tempol administered before radiation effectively protected the salivary glands from damage, which resulted in increased salivary secretion. In C3H mice, at doses of 15, 17.5, and 20 Gy, the decrease in salivary secretion was about 60, 70, and 80%, respectively. In mice treated with Tempol, much smaller values of the decrease in secretion were observed, i.e., 30, 50, and 70%, respectively [75]. Studies conducted on C3H mice have shown varying effectiveness of nitroxides in protecting their healthy cells. Nitroxides: Tempol (**53**), 3-CP (3-carbamoyl Proxyl, 3-carbamoyl-2,2,5,5-tetramethylpirrolidine-1-oxyl) (**65**), amine derivative (**62**), pyrrolidine derivative (**35**), and piperidine derivative (**36**) were administered in doses equal to MTD (maximum tolerated dose) 5 min before irradiation of the animal body with different doses of radiation. The most effective was nitroxide (**36**), for which LD50/30 was 11.47 Gy, while the value for the control group without radioprotector was only 7.9 Gy. Tempol and 3-CP were less effective, for which LD50/30 was 9.11 and 9.5 Gy, respectively. The studies conducted showed that the timing of nitroxide administration is very important. Nitroxide (**36**) administered not 5 min before but immediately after irradiation with a dose of 11 Gy did not provide any protection. The authors suggest that the protective role of nitroxides is related to the protection of bone marrow cells against ionizing radiation. Furthermore, pharmacokinetic studies using MRI have shown that nitroxide (**36**) is effective in redox imaging of the mouse brain. Moreover, (**36**) can be used in functional imaging of the myocardium. The obtained results suggest that nitroxide (**36**) is one of the most potent radioprotectors and can also be used as a contrast agent for functional imaging [76].

The most commonly used nitroxide was the hydrophilic Tempol, which easily penetrates biological membranes. It has been used as a radioprotector in vitro and in vivo. Tempol exhibits differential protection in normal and neoplastic tissues and has been used in oncological patients undergoing radiotherapy. Interestingly, local application of Tempol prevented alopecia that occurs during brain radiotherapy [77,78]. Although hydroxylamine did not show any radioprotection in vitro, its administration in vivo protected C3H mice from radiation. Tempol–H (hydroxylamine Tempol) is rapidly oxidized in vivo to Tempol. Tempol-H was administered to C3H mice before whole-body irradiation. The pharmacokinetics of Tempol-H were similar to Tempol alone after intraperitoneal injection. Only a small proportion of control mice survived 9 Gy, and none survived 10 Gy. However, after the administration of Tempol-H, there was a dramatic increase in the fraction of surviving mice. As with Tempol, over 60% of mice given Tempol–H survived 11 Gy, and over 10% survived thirty days after 13 Gy. Reduced nitroxides have also been shown to be radioprotective in vivo. However, Tempol causes significant hypotension, unlike other nitroxides, which have minimal effect [79]. Similar studies have been performed with several other nitroxides in C3H mice irradiated with a single dose of 9 Gy. Only 15% of the control group survived 30 days, while in the nitroxide groups, survival ranged from 35 to 100% depending on the nitroxide used. A small fraction of control mice survived irradiation at 8 and 9 Gy, none survived 30 days after 10 or 11 Gy. Mice given 3-carbamoyl-Proxyl (**65**) showed unchanged survival up to 9 Gy, and few survived 30 days after irradiation at 11 Gy, which is evidence that nitroxides provide good radiation protection [80]. Nitroxide JP4-039 (**30**) is a conjugate that accumulates in mitochondria and these organelles act as an antioxidant against reactive oxygen species (ROS) (Figure 5). To investigate the structure-activity relationships (SAR), new analogs with variable nitroxide fragments were prepared. It was shown that the synthesized analog labeled with a fluorophore accumulated in mitochondria. Spin-labeled analogs showed radioprotective and radiomitigating effects in 32Dcl3 cells [81]. JP4-039 showed mitochondrial localization and higher drug efficacy than Tempol. It was also shown that intraperitoneally administered JP4-039 reduced gamma-irradiated KM101 cell death (irradiated with 0 to 8 Gy) in vitro and prolonged survival of lethally irradiated (9.5 Gy) mice in vivo [82].

The mitochondrial-localizing hemi-GS-TEMPO conjugate (**32**) (Figure 5) was shown to exhibit radioprotective/mitigating effects in mouse embryonic cells and human bronchial epithelial BEAS-2B cells transformed with the hybrid adenovirus-12SV40 irradiated with γ rays. The authors suggest that this nitroxide was an effective electron scavenger and inhibited superoxide generation, as well as inhibited cardiolipin oxidation in mitochondria, which prevented the release of proapoptotic factors from mitochondria and delayed irradiation-induced apoptosis. Furthermore, the hemi-GS-Tempo conjugate initiated cell cycle arrest in the G2/M phase, which may contribute to cell protection [83].

The skin is one of the largest human organs and is the first line of defense against external factors such as radiation and various chemicals. UV radiation can initiate skin damage, which includes photoaging and skin carcinogenesis. In turn, ionizing radiation used in radiotherapy can cause radiation dermatitis. Radiation initiates oxidative stress, which is responsible for skin damage, and among the cellular organelles, mitochondria are particularly sensitive to oxidative stress. The process of apoptosis triggered by mitochondria is involved in cell and tissue damage resulting from radiation exposure. It has been shown that in irradiated mice at a dose of 35 Gy (γ radiation), after 21 days of irradiation, characteristic burns with severe desquamation and ulceration at the irradiation site appeared. In contrast, in mice treated with JP4-039 the skin was intact, with minimal erythema. JP4-039 nitroxide has been shown to prevent and ameliorate radiation-induced skin damage, thereby limiting skin inflammation, loss of barrier function, and fibrosis. Additionally, limiting damage leads to reduced apoptosis, maintaining the antioxidant potential of the skin, and limited oxidative damage to DNA, proteins, and other macromolecules by oxidative stress [84].

Studies conducted on the retina of the eye showed that intravitreally injected JP4-039 inhibited apoptosis and inflammatory cell migration in irradiated mouse retina (Figure 5). Further studies may complete the safety profiling as well as the possibility of using this nitroxide in patients with radiation retinopathy [85]. Normal tissue-specific radioprotection of the oral cavity was studied in radiosensitive Fanconi anemia mice with oral tumors, using mitochondrial JP4-039. JP4-039 modulated radiation-induced transcript growth in normal tissue, as well as ameliorated mucosal ulceration and reduced the decrease in endogenous antioxidants in oral tissue. Such properties were not demonstrated by 4-aminoTempo (**55**). However, nitroxide was an effective radioprotector of normal tissue but did not protect tumors from radiation [86].

**Figure 6:**
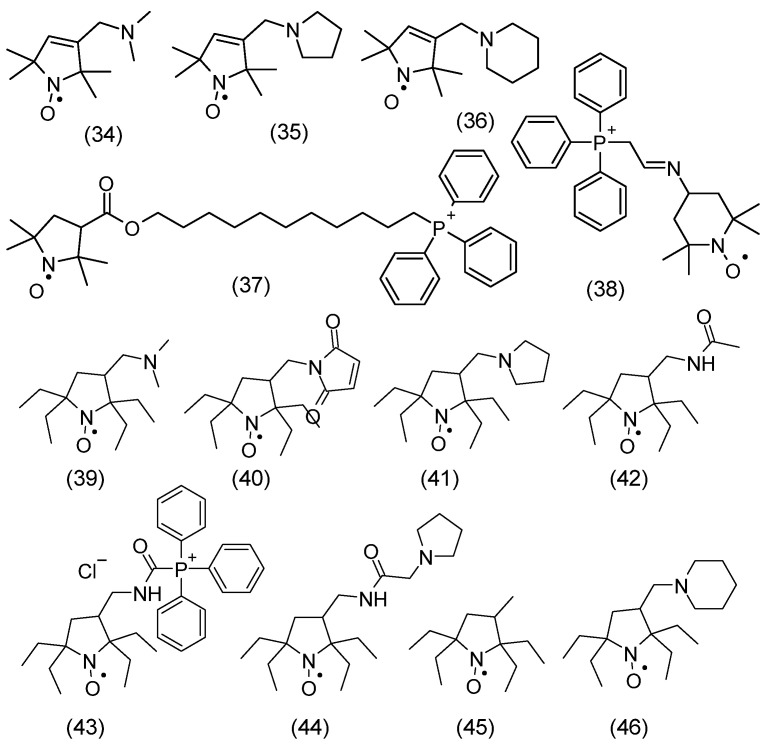
Nitroxides used as radioprotectors against ionizing radiation: dimethylamine derivative (**34**), pyrrolidine derivative (**35**), piperidine derivative, (**36**) Mito-CP (**37**), TPEY-Tempo (**38**) and their spatially shielded counterparts **39**–**46**.

TPEY-Tempo nitroxide (**38**), a triphenylphosphonium derivative of Tempo, is characterized by strong compartmentalization in mitochondria, and therefore greater protection compared to healthy cells (Figure 6). Studies have shown that half an hour of preincubation of normal BB19 brain cells and T98G glioma cells with TPEY-Tempo provided strong protection only to the former. TPEY-Tempo (**38**) proved to be an effective radioprotector in normal brain cells and, at the same time, increased the sensitivity of tumor cells to γ radiation, which was also observed in another glioma cell line U87MG. The above properties of nitroxide were confirmed by examining the level of released cytochrome c and caspase activity [69]. The efficacy of nitroxide JP4-039 was also confirmed in vivo in mice. JP4-039 protects against esophagitis, which occurs in radiotherapy for lung cancer. In mice receiving nitroxide in liposomal form before chest irradiation (29 Gy once or 11.5 Gy four times), esophagitis did not occur. Mice receiving nitroxide survived significantly longer than the control group. In addition, JP4-039 protected not only esophageal cells but also cells located in the blood, bone marrow, and liver, protecting them from the harmful effects of radiotherapy. Interestingly, JP4-039 also penetrated lung cancer cells, showing no protection against them [87]. Cell lines derived from patients with Fanconi anemia are a valuable model for research on the toxicity of ionizing radiation. One of the features of this disease is high sensitivity to the development of tumors and high sensitivity to γ radiation, which is caused by impaired DNA repair mechanisms and excludes the possibility of using radiotherapy. In vivo and in vitro studies have shown that the use of JP4-039 nitroxide contributed to the protection of healthy cells from the toxic effects of γ radiation [70,88,89]. Nitroxide prevented ulceration of mucous membranes and protected against damage to bone marrow stromal cells and oral tissues. It inhibited cardiolipin oxidation, which prevented cytochrome c from being released into the cytosol and apoptosis from being initiated. Studies on the expression of genes encoding proinflammatory proteins and proteins activated in response to oxidative stress and DNA damage proved the radioprotective activity of nitroxide JP4-039 [70,88,89].

### 3.4. Nitroxides in the Protection of Cells and Organs by Hypoxia/Reperfusion

Excessive generation of reactive oxygen species (ROS) after reperfusion is considered to be an important factor related to the pathophysiology of neurological damage induced by cardiac arrest (CA). However, the use of antioxidant therapies in clinical trials has been unsuccessful. XJB-5-131 (**30**) was shown to effectively inhibit monolyso-CL production and lipid mediator release, as well as CA-induced biochemical and behavioral abnormalities compared with control rats (Figure 5). Mitochondrial-targeted nitroxides prevented cardiolipin oxidation and subsequent hydrolysis; furthermore, they inhibited caspase activation and improved neurocognitive stimuli after cardiac arrest. These data demonstrated that calcium-independent CL oxidation and subsequent hydrolysis revealed a new unidentified pathogenic mechanism of ischemia/reperfusion-induced brain injury [90]. The release of ROS in the electron transport chain in mitochondria has a major role in the pathogenesis of aging as well as age-related diseases. Aging often leads to cardiovascular diseases, especially myocardial infarction, which is the cause of chronic disability and mortality in the elderly. Aging hearts are very sensitive to stress, including oxidative stress, which is associated with, for example, ischemia/reperfusion (I/R). It has been shown in human and animal populations that older hearts are more sensitive to myocardial injury after I/R than younger ones [91,92]. Studies in adult and aged rats have shown that the nitroxide XJB-5-131 (**30**), a mitochondrial ROS and electron scavenger, improved recovery from ischemia in aged hearts compared with the group that did not receive nitroxide. Furthermore, the respiration rates in complexes I, II, and IV of mitochondria isolated from nitroxide-treated aged hearts were 57%, 25%, and 28%, respectively, higher than controls. It can be assumed that mitochondrial dysfunction is a major factor in the pathogenesis of the aging heart [93].

Sublytic concentrations of hydrogen peroxide, combined with the nitric oxide (NO^•^) donor SNAP (S-nitroso-N-acetyl-d,l-penicillamine), initiated oxidative/nitration stress via activation of the p38 MAPK and p53 cascades. DNA damage and tyrosine nitration in proteins were also observed. Studies using six antioxidants, including SOD, CAT, Tempo, N-acetylcysteine, dimethylthiourea, and uric acid, showed that Tempo (**52**) was the most effective antioxidant that inhibited H_2_O_2_ and SNAP-induced changes, i.e., activation of stress proteins p38 MAPK and p53 and the generation of ROS, nitric oxide, and peroxynitrite, including the initiation of double-strand DNA breaks and tyrosine nitration in proteins [94].

**Figure 7:**
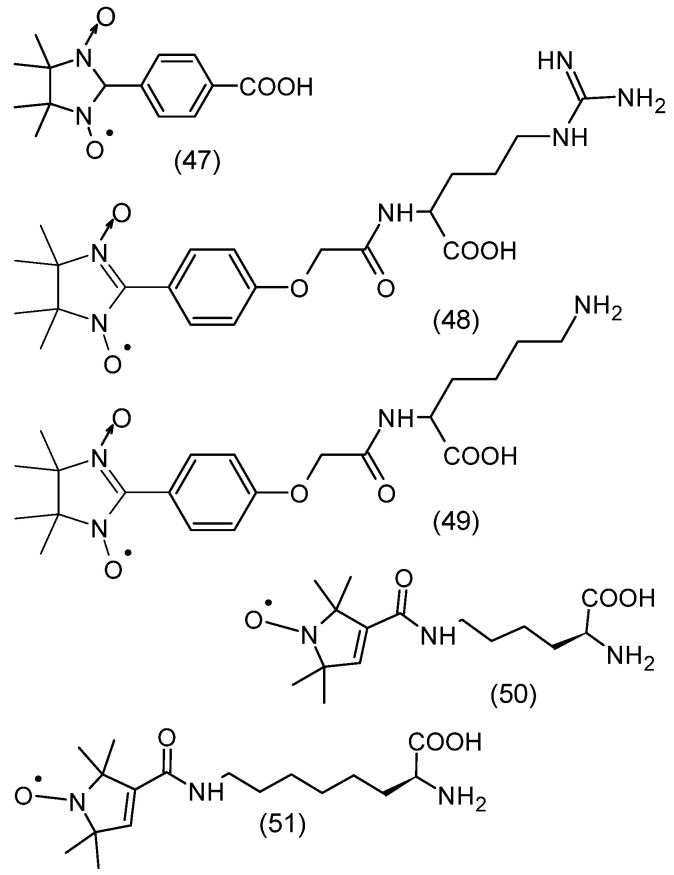
cPTIO (**47**), amino acid derivatives of nitronyl-nitroxide (**48** and **49**) and amino acid derivatives of pyrroline (**50**), (**51**).

Nitronyl nitroxide, 2-(4-carboxyphenyl)-4,4,5,5-tetramethylimidazoline-1-oxyl-3-oxide (cPTIO) is useful in monitoring nitric oxide (Figure 7). The change in the structure of nitronyl nitroxide upon NO^•^ uptake to imino nitroxide is associated with a change in the hyperfine structure of the EPR spectrum and therefore can be monitored in real time. However, in biological systems, nitroxides are reduced to diamagnetic compounds that do not emit an EPR signal. Nevertheless, cPTIO (**47**) can be an inhibitor in some reactions involving NO^•^, but not all [95]. An example is the reaction of NO^•^ with superoxide with a reaction rate constant 6.7 ± 0.9 × 10^9^ M^−1^ s^−1^, which is about 3 times faster than the scavenging of superoxide by SOD.

Nitronyl nitroxide amino acid derivatives (NNR (**48**) and NNK (**49**)) were shown to effectively scavenge NO^•^ in an acetylcholine-induced vasodilation test (Figure 7). Furthermore, NNR (**48**) showed a protective effect on liver injury induced by ischemia–reperfusion in an in vivo rat model. The mechanism of liver injury was related to oxidative stress, which was abolished by nitroxide [96]. Tempol administered intravenously to rats and rabbits in models of myocardial infarction reduced the size of infarcts by 30–40% [97]. Tempol was also shown to reduce the size of cerebral infarction in rats initiated by occlusion of the middle cerebral artery. Studies revealed that after reperfusion, the infarcted area was significantly smaller compared to the animal control group [98].

Renal tubular epithelial cell (TEC) damage and subsequent cell death initiate acute kidney injury and subsequent chronic kidney disease (CKD). The cause of kidney disease is ferroptosis, which occurs in tubular epithelial cells and is associated with the expression of the tubular pro-ferroptosis gene. It was shown that XJB-5-131 nitroxide, which had a high affinity for TEC, reduced renal injury and inflammation induced by I/R in mice by specifically inhibiting ferroptosis. It showed that genes involved in ferroptosis were especially expressed in tubular epithelial cells after I/R, indicating that ferroptosis is important in renal tubular injury. Therefore, inhibition of ferroptosis by nitroxide may be a promising therapeutic method for protecting renal tubular cells in CKD [99].

### 3.5. Nitroxide Reduction/Bioreduction

One of the applications of nitroxides is their use as contrast agents for magnetic resonance imaging and as spin probes and labels in EPR spectroscopy. However, their rapid reduction in biological systems to hydroxylamines greatly limits their use. Therefore, methods for the synthesis of nitroxides with increased stability to reducing agents in vivo and in vitro have been developed. One of the most important reducing agents occurring in cells and tissues is ascorbic acid (ascorbate, AscH^−^). The more sterically shielded nitroxide is another important factor in increasing the stability of nitroxide to ascorbate [100,101,102]. This applies to the reduction of piperidine, pyrroline, pyrrolidine, imidazoline, and imidazolidine derivatives and other nitroxides presented in Figure 1. The introduction of ethyl groups instead of methyl groups leads to an increase in the stability of nitroxides to in vivo and in vitro reductions. Interestingly, at high concentrations of ascorbic acid, hydroxylamines are reoxygenated to nitroxides by ascorbyl anion radical (AscH^−^) and dehydroascorbic acid (DHA). For example, the rate constants of the one-electron reduction of tetraethyl-substituted nitroxides by ascorbate ranged from 2.65 × 10^−6^ to 10^−5^ M^−1^s^−1^ and were much lower compared to the values for nitroxides containing methyl substituents (k > 10^−4^ M^−1^s^−1^) [103]. Although glutathione itself does not lead to nitroxide reduction, when it is present, a significant modification of nitroxide reduction by ascorbate is observed. This process is related to the reduction of ascorbyl radicals by GSH, and the rate constant of this reaction is 10 M^−1^s^−1^. In turn, the rate constants of bimolecular reduction by ascorbate are significantly higher for tetramethyl piperidine derivatives than for pyrroline and pyrrolidine. For example, for Tempo and Tempol they are 3.5 M^−1^s^−1^ and 7 M^−1^s^−1^, respectively [104]. In turn, for five-membered pyrrolidine derivatives, 0.07–0.3 M^−1^s^−1^, and imidazolidine, 0.85 M^−1^s^−1^ for 10 [105]. On the other hand, the presence of a double bond in position 3 of the pyrroline and imidazoline rings leads to an increase in the rate of reduction by ascorbate, the rate constants being 0.64–1.6 M^−1^s^−1^ and 5.6 M^−1^s^−1^ for 9, respectively [103,105]. The presence of a negative charge is an additional factor stabilizing the nitroxide against reduction by the negatively charged ascorbate [106]. An example is carboxy-Proxyl (**64**) as the most resistant to reduction by ascorbate (k = 0.1 M^−1^s^−1^).

During the reduction of nitroxide by ascorbate, hydroxylamine and ascorbyl anion radical are formed. However, the disappearance of AscH^−^ causes a shift in the equilibrium towards hydroxylamine through multi-stage mechanisms [100]. The introduction of glutathione to the reaction environment leads to the capture of ascorbyl anion radical or dehydroascorbate, which is formed as a result of the disproportionation of the ascorbate radical [100,107]. The introduction of GSH causes a higher conversion of nitroxide to hydroxylamine. This effect is more visible at higher ascorbate concentrations. The greater resistance to reduction of tetraethyl-substituted nitroxides compared to spirocyclohexyl groups (nitroxide 71–74) may be related to the increased steric shielding of the nitroxide group, as shown in the examples of space-filling X-ray structures [108]. Similarly to nitroxides containing four methyl groups, tetraethyl-derivatives of nitroxides showed inhibitory effects on lipid peroxidation, but showed low antiproliferative activity in HepG2 and HUVEC cells and had no effect on blood pressure in animals [109]. The advantage of tetraethyl-substituted piperidine radicals was their stability, for 20 min, after intravenous injection in mice as measured by EPR signal intensity, indicating that they are stable to bioreduction by ascorbate while retaining antioxidant and paramagnetic tracer/contrast agent properties. Therefore, they may be useful in identifying oxidative stress foci in vivo using redox-based imaging techniques [109].

**Figure 8:**
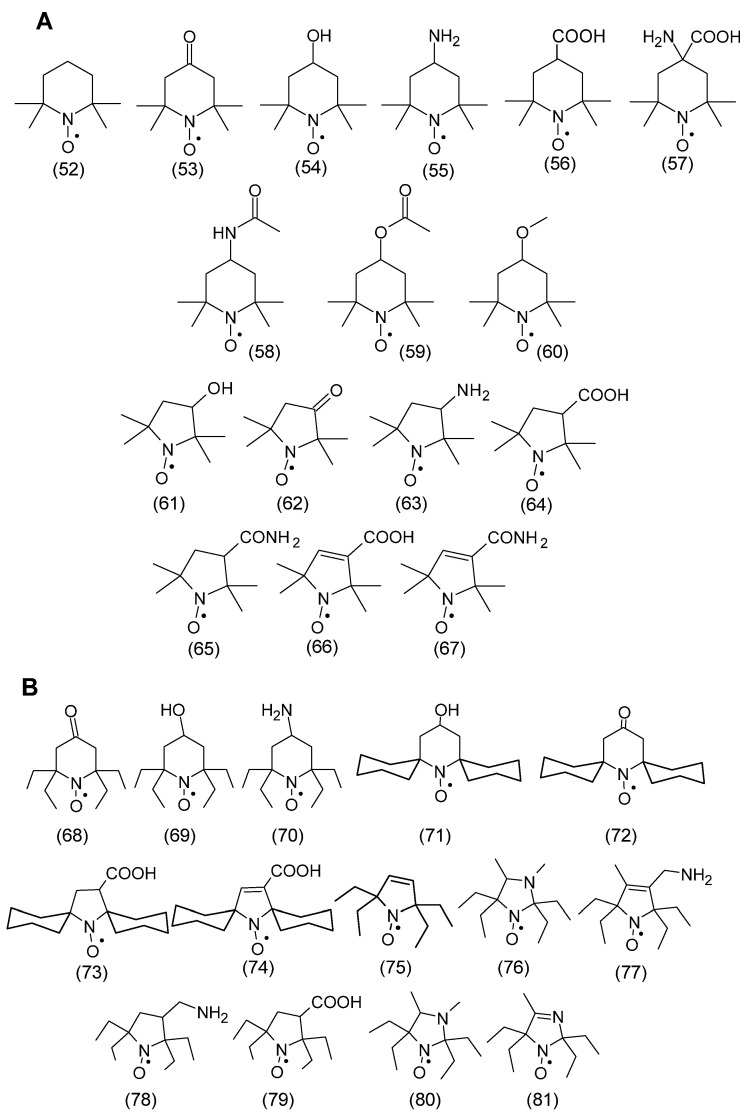
(**A**). The most well-known classical nitroxide: Tempo (**52**), Tempone (**53**), Tempol (4-hydroxyTempo) (**54**), Tempamine (4-aminoTempo) (**55**), 4-carboxyTempo (**56**), aminoacidTempo (**57**), Tempace (**58**), acyl ester of Tempol (**59**), 4-methoxy-Tempo (**60**), 3-hydroxy-Proxyl (**61**), 3-oxo-Pyrrolin (**62**), 3-amino-Proxyl (**63**), 3-carboxy-Proxyl (**64**), 3-carbamoyl-Proxyl (**65**), 3-carboxy-Pyrrolin (3-CP) (**66**), 3-carbamoyl-Pyrrolin (**67**). (**B**). Classical nitroxide equivalents sterically shielded: piperidine (**68**–**72**); pyrrolidine (**73**,**78**,**79**) and pirroline (**74**,**75**,**77**); imidazolidine (**76**,**80**) and imidazoline (**81**).

It has been shown that sterically shielded pyrrolidine derivative nitroxides containing four ethyl groups are characterized by the highest stability in biological systems compared to other tetramethyl nitroxides [110]. Since 3-carboxy-Proxyl (**64**) showed the highest stability to reduction, the nitroxide, 3-carboxy-2,2,5,5-tetraethylpyrrolidine-1-oxyl (C-TETPO) (**79**) belongs to the even more resistant to reduction (Figure 8). This nitroxide was reduced more slowly in the cytosolic extract than the trityl (triphenylmethyl) radical [111,112]. The second-order rate constant of C-TETPO with ascorbate in the presence of GSH was 3.3 × 10^4^ M^−1^s^−1^ and was similar (range 2.4–4.1 × 10^4^ M^−1^s^−1^) to other teraethylpyrrolidine derivatives used as spin labels [110]. Two new nitroxyl radicals 3-aminomethyl-2,2,5,5-tetraethylpyrrolidine-1-oxyl (AM-TETPO) (**78**) and a TETPO derivative containing a triphenylphosphonium substituent (Mito-TETPO) (**43**), were shown to be highly stable in mouse blood, where the content of reducing agents is low. No reduction of AM-TETPO (**78**) was observed within 30 min of the experiment. However, a decrease in Mito-TETPO (**43**) concentration was observed by about 10% in the first 10 min, reaching a plateau thereafter. A slightly faster decay of the Mito-TETPO EPR signal may be related to its reduction inside blood cells [110]. In turn, a much greater difference in the reduction of both nitroxides was revealed in homogenates of various mouse organs. A faster decay of the Mito-TETPO nitroxide signal compared to AM-TETPO was recorded in brain and liver homogenates, which was probably related to the directed accumulation of Mito-TETPO in intact mitochondria [110]. On the other hand, the decrease in the AM-TETPO EPR signal by 50% within 30 min could not be caused by its reduction, considering the low physiological concentrations of ascorbate [113]. The disappearance of both nitroxides may be related to their destruction by cytochrome P450, which would be consistent with the observations of Babic et al. [114]. Cytochromes P450 are found in the liver, brain, and kidneys, where they participate in local metabolism [114,115,116]. In addition, they are also found in mitochondria [117], which may result in the faster degradation of Mito-TETPO (**43**) compared to AM-TETPO (**78**).

### 3.6. Nitroxide Actions in Cells and Tissues

Mito-carboxy-Proxyl nitroxide (Mito-CP), targeting mitochondria, inhibited oxidative stress (OS) and apoptosis in bovine aortic endothelial cells (BAEC) (Figure 6). OS was initiated by the glucose/glucose oxidase (Glu/GO) system, as well as lipid peroxide. Furthermore, Mito-CP inhibited cytochrome c release and caspase-3 activation, complex I and aconitase inactivation, as well as transferrin receptor (TfR) expression and ^55^Fe uptake by mitochondria, which restored mitochondrial membrane potential and proteasome activity. In contrast to Mito-CP, carboxyProxyl (CP) did not protect cells against OS and superoxide-initiated apoptosis [118].

Nitroxides XJB-5-131 and JP4-039, which attenuate lipid peroxidation, were shown to prevent ferroptotic (non-apoptotic) cell death in HT-1080, BJeLR, and panc-1 cells. Their biological activity was related to relative lipophilicity, showing a significant contribution of intramitochondrial lipid peroxidation to ferroptosis. It can be assumed that the prevention of mitochondrial lipid oxidation may be one of the therapeutic applications in tissue damage caused by ischemia/reperfusion, acute kidney injury, and other pathologies associated with ferroptotic cell death pathways [119]. Tuberculosis is a chronic inflammatory disease caused by the bacteria *Mycobacterium tuberculosis*. The bacterium is resistant to most antibiotics but also to oxidative killing. In addition, *M. tuberculosis* primarily exploits the production of host oxidants to induce phagocyte death, thereby allowing the bacteria to spread. The use of 4-methoxy-Tempo (**60**) in a zebrafish-M and the marinum infection model inhibited mitochondrial ROS production and reduced infection-induced cell death, which allowed for the inhibition of infection (Figure 8). Persistent hypoxia in the microenvironment of infection may sensitize *Mycobacterium tuberculosis* to nitroxide killing both in vitro and in vivo [120]. In vitro studies have shown that XJB-5-131 inhibited apoptosis and also improved the survival of mouse embryo-derived cells [121]. Oxidative damage and mitochondrial dysfunction are comorbidities of aging, as well as age-related neurodegenerative diseases, including Huntington’s disease (HD). XJB-5-131 has been shown to ameliorate the disease phenotype in a mouse model of Huntington’s disease and improve mitochondrial function [122]. XJB-5-131 has also been shown to prevent ileal mucosal barrier dysfunction and tissue ischemia by inhibiting peroxidation of the mitochondrial phospholipid cardiolipin in a rat model of hemorrhagic shock (HS). Furthermore, it also attenuated HS-induced activation of proapoptotic caspases 3 and 7 in the ileal mucosa. Intravenous administration of the compound significantly prolonged survival in rats subjected to severe blood loss [123].

**Figure 9:**
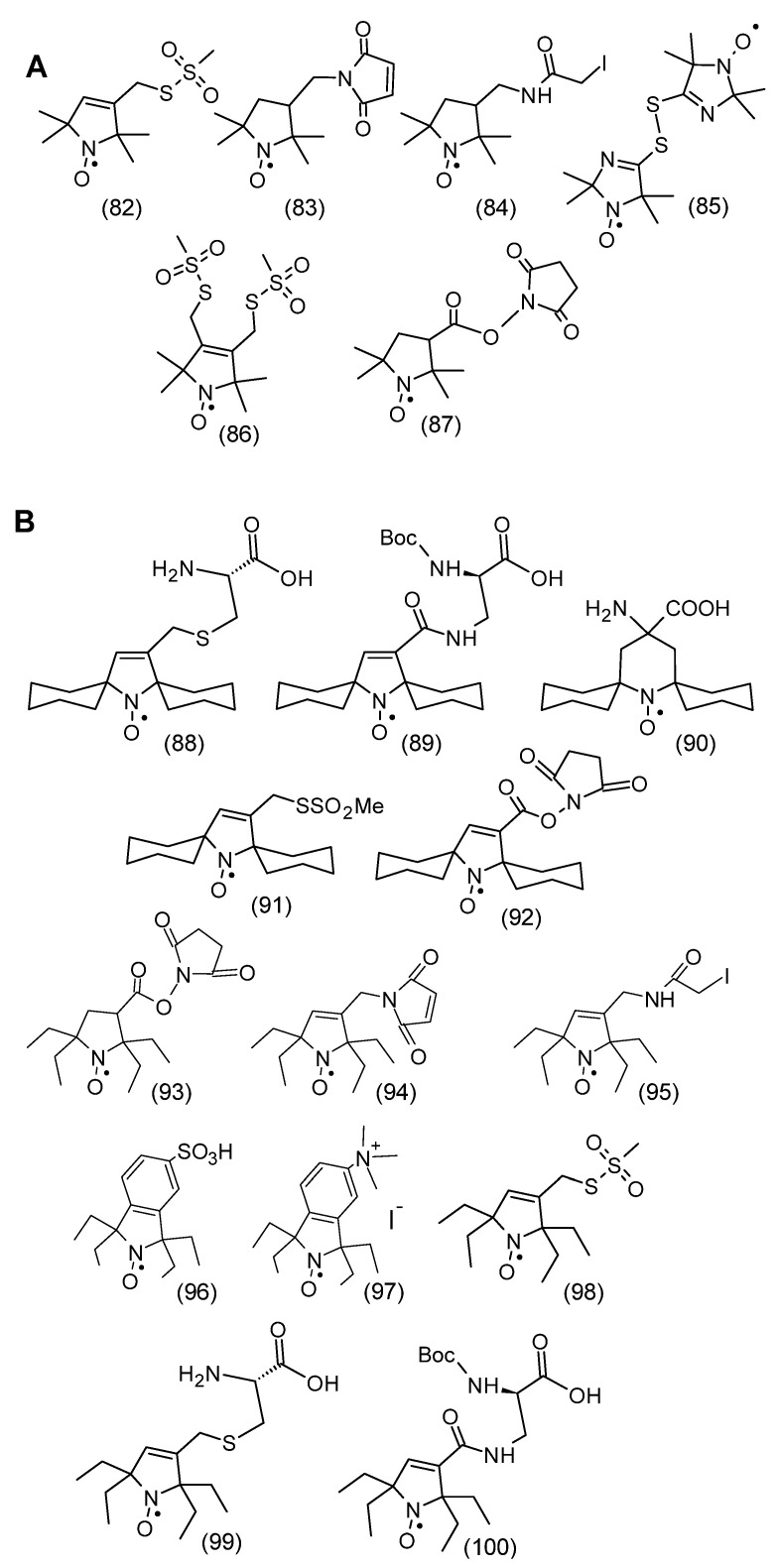
(**A**) The most commonly used spin labels (**82**–**87**) and (**B**) their spatially shielded counterparts (**88**–**100**).

Analogously to the spatially shielded typical nitroxides shown in Figure 8, spatially shielded spin labels were synthesized. Figure 9A shows classical spin labels reacting with thiol, amino, and hydroxyl groups of proteins and peptides. In turn, Figure 9B shows similar spatially shielded spin labels.

### 3.7. Nitroxides in Erythrocytes

Nitroxides in erythrocytes have been relatively best. In our previous work, we showed that nitroxides derived from 2,2,6,6-tetramethylpiperidine (Tempo (**52**), Tempol (**54**), and Tempamine (**55**) and nitroxyl derivatives of 2,2,5,5-tetramethopyrrolidine and 2,2,5,5-tetramethopyrrolidine modulate the antioxidant system in human red blood cells. These derivatives differed in the presence of different substituents in the piperidine ring position and the 3-position of the pyrroline and pyrrolidine rings. Piperidine nitroxides have a chair structure of the heterocyclic ring, while the structure of pyrroline derivatives is flat, which is caused by the presence of a double bond in the ring. Only the nitroxyl group “protrudes” above the plane of the ring. However, the heterocyclic ring structure of pyrrolidine nitroxides is not planar, and the atoms forming the ring do not lie in one plane. Interestingly, the half-life of piperidine nitroxides in red blood cells is about 1 h. In contrast, the half-life of five-membered nitroxides was at least five to six times longer.

In cells, nitroxides undergo single-electron reduction to hydroxylamines by ascorbic acid, NADPH, and reducing enzymes [37]. The use of -SH group inhibitors, such as Hg(II) ions, p-chloromercuric benzoic acid, and N-ethylmaleimide) inhibited the rate of nitroxide reduction in erythrocytes [124,125]. On the other hand, the use of ionizing radiation accelerated the rate of nitroxide reduction in erythrocytes [126]. Although glutathione (GSH) does not reduce nitroxides even at a 100-fold excess in a direct reaction, in cells [127], nitroxides can also be reduced by thiols [128,129]. Nitroxides led to a decrease in the level of glutathione in red blood cells [130]. Piperidine nitroxides (Tempo, Tempol, and Tempamine) and pyrrolidine nitroxides caused a decrease in the GSH concentration below 10%, while pyrroline nitroxides reduced GSH only by 55–60%, which indicates a significant effect of the heterocyclic ring structure [130]. Other authors also observed a decrease in glutathione concentration caused by Tempo in RBCs [131]. Furthermore, nitroxides led to a decrease in the activity of glutathione-dependent enzymes such as glutathione peroxidase (GPx), glutathione sulfate transferase (GST), and glutathione reductase (GRx) in RBCs [132]. In contrast, nitroxides did not affect the total glutathione pool (GSH + GSSG) in RBCs, suggesting that glutathione did not form conjugates with nitroxides that could be transported out of the cell. The reduction of nitroxides in RBCs occurs mainly through the reaction with ascorbic acid (AA) and not through the reaction with glutathione. The decrease in glutathione concentration in RBCs is probably related to the regeneration of DHA (dehydroascorbate) to ascorbate by GSH [107]. DHA is a product that is formed as a result of the reduction of nitroxides by ascorbic acid. The most significant decrease in AA concentration in erythrocytes occurred after treatment with Pyrrolide (3-Carbamoyl-Proxyl). The decrease in ascorbic acid concentration in RBCs was greater in the case of six-membered nitroxides than in the case of five-membered nitroxides. Nitroxides Tempol and Tempamine, but also 3-Carbamoyl-Proxyl, which reduced the concentration of ascorbic acid in red blood cells the most [130]. Comparing the midpoint potential (mV) with the normal hydrogen electrode, small differences were found in the normal potentials for Tempol, Tempamine, and Carboxy-Pyrrolid [133]. In the model conditions, the rate of nitroxide reduction depended on the concentration of ascorbic acid and on the presence of oxygen. Interestingly, in anaerobic conditions, the rate of reduction was much higher than in aerobic conditions [127]. Moreover, it was found that the rate of reduction of piperidine nitroxides depended on the substituent at position 4. The presence of the substituent affected the rate of reduction of piperidine nitroxides, for example, Tempol and Tempace (4-acetamido-2,2,6,6-tetramethylpiperidine-1-oxyl) were reduced faster by AA than Tempo, which is correlated with the electronegativity of the functional groups. Nitroxides induced an increase in the activity of superoxide dismutase in erythrocytes. The highest increase in activity (approx. 75%) was observed for Pyrrolide and the lowest for Carboxy-Pyrrolide (approx. 42%). The increase in SOD activity may indicate overproduction of superoxide anion in erythrocytes after the action of nitroxides. In the case of LDH, only Tempol significantly reduced the activity of this enzyme. However, none of the five-membered nitroxides had any effect on the activity of LDH. Nitroxides also cause the oxidation of hemoglobin in red blood cells. Among the six-membered nitroxides, Tempo and Tempol showed such activity, while Tempamine was inactive. Among the five-membered nitroxides, only pyrroline nitroxides were effective in oxidizing Hb to MetHb. The oxidation of Hb to MetHb by Tempo was also described in another paper [131]. In turn, indoline and quinoline nitroxides (1,2-dihydro-2-ethyl-2-phenyl-3 H -indole-3-phenylimino-1-oxyl and 1,2-dihydro-2,2-diphenyl-4-ethoxyquinoline-1-oxyl, respectively) induced hemolysis of trout erythrocytes but also oxidized Hb to MetHb [134]. Finally, we determined the effect of nitroxides on the initiation of oxidative stress in erythrocytes. Despite their prooxidant properties, nitroxides did not cause lipid peroxidation or protein oxidation. Interestingly, piperidine nitroxides induced an increase in the level of thiol groups in plasma membrane proteins and hemolysate [135].

### 3.8. Nitroxide in Other Cells

Nitroxides were also reduced to hydroxylamines in immortalized B14 fibroblasts. Large differences in the rate of reduction of piperidine nitroxides were demonstrated. Tempo was reduced twice as fast as Tempamine and four times faster than Tempol and Tempace. Similarly to erythrocytes, pyrroline and pyrrolidine nitroxides were reduced several times faster than piperidine nitroxides. Interestingly, piperidine nitroxides effectively reduced oxidative stress initiated by hydrogen peroxide and doxorubicin in these cells [136]. Recently, Tempo was shown to slow down the growth rate of wild-type strains and isogenic BY4741 mutants with impaired response to oxidative stress (sod1Δ, sod2Δ, yap1Δ). Nitroxide also slowed down postmitotic cell aging in the yeast population. Yeast cells treated with nitroxide were characterized by significantly increased levels of metabolism in wild-type and sod2Δ strains. Interestingly, Tempo also had a genoprotective effect, protecting DNA from damage by reducing the number of double-stranded DNA breaks in cells. In addition to its protective effect on yeast, Tempo also showed toxic effects, especially on active mitotic cells, leading to cell cycle arrest [137].

While the antioxidant mechanism of nitroxides has been well studied, much less is known about their prooxidant action. The effect of Tempol on inhibition of cell proliferation and its effect on induction of apoptosis in the presence of cisplatin (DDP) in ovarian cancer cells was studied. It was shown that the proliferation rate of OVCAR3 and SKOV3 cells was inhibited by Tempol at concentrations of 1.5 and 1 mM, respectively, compared to the control group. In the case of combination therapy, a significant inhibition of OVCAR3 cell proliferation was observed compared to the use of DDP alone. It was also shown that combination therapy increased the percentage of early apoptotic cells in OVCAR3 cells compared to DDP single treatment. Furthermore, combination therapy led to a significant decrease in the Bcl-2:Bax expression ratio compared to DDP alone. It was shown that combination therapy significantly increased ROS production in cells compared to DDP alone. These data indicated that Tempol potentiated the effect of DDP on initiating apoptosis in OVCAR3 cells [138].

The cytotoxicity and genotoxicity of Tempo in mammalian cells were investigated using the mouse lymphoma assay (MLA) and the in vitro micronucleus assay. 3 mM Tempo induced significant cytotoxicity and marginal mutagenicity in MLA; in the absence of metabolic activation (S9). Treatment of mouse lymphoma cells with 1–2 mM Tempo led to dose-dependent reductions in relative overall growth and increased mutant frequency. In contrast, treatment of human lymphoblastic cells with TK6 increased the frequency of micronuclei (a marker of clastogenicity) and hypodiploid nuclei (a marker of aneugenicity) in a dose-dependent manner. Greater responses were obtained in the presence of S9. Tempo initiated ROS production and decreased glutathione levels in mouse lymphoma cells. Most Tempo-initiated mutants had a loss of heterozygosity at the Tk locus, with allelic loss ≤ 34 Mbp. These results showed that Tempo is mutagenic in MLA and induces micronuclei and hypodiploid nuclei in TK6 cells. Oxidative stress initiated by Tempo may be responsible for part of the genotoxicity induced by this nitroxide in both cell lines [71].

The influence of the structure of different nitroxides on the inhibition of oxidative damage to two proteins (albumin (HSA) and perlecan, a proteoglycan of the extracellular matrix) was determined. Without nitroxides, these proteins were strongly modified by the MPO-H_2_O_2_-Cl^−^ complex. The ability to recognize the core of the perlecan protein by CSI-076 was observed. In the case of HSA, however, the loss of Met and histidine residues was observed. The use of nitroxides (4-aminoTempo and Tempol) strongly limited protein modifications, but, e.g., 4-carboxyTempo (4-carboxyl-2,2,6,6-tetramethylpiperidine-1-oxyl) was ineffective. Hydroxylamines (TempoH and 4-aminoTempoH) and secondary amine derivatives were similarly ineffective [64]. On the other hand, hydroxylamines revealed significant inhibition of neutrophil-mediated HOCl production. Interestingly, 4-aminoTempo reduced HOCl production by neutrophils much more than 3-aminoProxyl. As with all other compounds that are redox active within biologically compatible limits, nitroxides also exerted prooxidant effects [63,139].

### 3.9. Applications of Nitroxides in Experimental Animals

There is still no effective therapy for age-related osteoarthritis. Some hopes are associated with nitroxides, which could alleviate the progression of the disease by inhibiting chondrocyte ferroptosis. It was shown that nitroxide XJB-5-131 significantly inhibited t-BHP-induced ferroptosis symptoms, such as ROS release, lipid peroxidation, and Fe(II) accumulation. Furthermore, nitroxide reduced ferroptosis-initiating factors (Ptgs2, Pgd, Tfrc, Atf3, Cdo1) and simultaneously restored the expression of ferroptosis suppressors (Gpx4, Fth1). XJB-5-131 increased the expression of anabolic cartilage markers and reduced the expression of catabolic cartilage markers. Intra-articular injection of XJB-5-131 significantly slowed down the expression of Cox2 and Mmp13, while increasing the expression of Col2a1, Gpx4, and Fth1 in the articular cartilage of mice initiated by destabilization of the medial meniscus (DMM). Pebp1 was also shown to be a possible target of XJB-5-131. The application of a specific Pebp1 antagonist, Locostatin, significantly reduced the antiferroptotic and chondroprotective effects of XJB-5-131 (Figure 5). Nitroxide XJB-5-131 showed a significant protective effect against ferroptosis in mouse chondrocytes initiated by t-BHP in vitro and in vivo in a mouse model of osteoarthritis induced by DMM surgery by restoring the expression of Pebp1 [140]. XJB-5-131 could likely be a potential drug used in osteoarthritis therapy.

### 3.10. Nitroxides in Neurodegenerative Diseases

**Figure 10:**
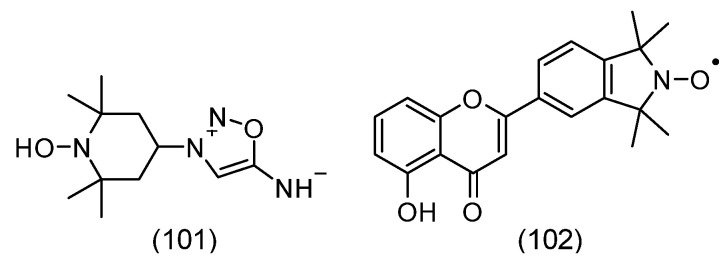
Hybrid molecule of Tempol (SA-2) (**101**) and spin-labeled flavonoid (**102**).

The generation of ROS is important in the pathogenesis of neurodegenerative diseases of the eye, such as glaucoma, age-related macular degeneration (AMD), and ischemic stroke of the eye. The increased generation of ROS and their accumulation occur with the progression of these diseases. Therefore, new therapeutic and prophylactic strategies based on exogenous antioxidants are sought. Recently, new synthetic therapeutic antioxidants have been sought that target ROS in neurodegenerative diseases. There is some hope in the use of new-generation hybrid Tempol derivatives, which may have new neuroprotective possibilities for the eye. Such a hybrid molecule SA-2 consists of a NO donor and an antioxidant part consisting of nitroxide (Tempol) (**54**) (Figure 8). The nitroxide part is supposed to capture the superoxide radical, thus preventing the production of peroxynitrite and depletion of NO bioavailability. In turn, the release of physiological concentrations of NO from the syndonimine fragment can activate soluble guanylate cyclase, releasing cGMP. Such action leads to relaxation of the trabecular meshwork, causes a decrease in intraocular pressure, and has a neuroprotective effect. Such compounds could reduce oxidative stress in the retina and optic nerve, preserving retinal ganglion cells and trabecular meshwork and protecting against optic nerve damage, which could alleviate irreversible blindness associated with these diseases [141]. However, the use of hybrid molecules is not always successful. Oxidative stress caused by an imbalance in homeostasis is associated with the progression of many neurodegenerative diseases. The use of appropriate antioxidants may be a promising option to slow down neurodegeneration. Using a hybrid pharmacological action, when nitroxides were combined with natural antioxidants, such as flavonoids, can increase their antioxidant potential (Figure 10). However, the evaluation of the superoxide radical scavenging capacity showed reduced antioxidant activity of hybrids compared to their components [142].

Excessive glutamate release is the main cause of retinal ganglion cell death in glaucoma. Cannabinoids are one of the effective drugs that protect neuronal cultures from glutamate-initiated death. Retinal excitotoxicity was initiated by intravitreal injection of N-methyl-D-aspartate (NMDA) in rats administered Tempol and N-omega-nitro-L-arginine methyl ester (L-NAME, NOS inhibitor), the psychotropic drug delta-9-tetrahydroxycannabinol (THC), or cannabidiol (CBD). NMDA initiated dose- and time-dependent nitrite/nitrate accumulation, lipid peroxidation, and tyrosine nitration, as well as dose-dependent apoptosis and loss of inner retinal neurons. Application of L-NAME or Tempol protected retinal neurons from damage and proved the involvement of peroxynitrite in retinal neurotoxicity. Neuroprotection by THC and CBD was the effect of inhibition of peroxynitrite [143]. Retinal ganglion cell atrophy occurs in chronic eye diseases such as glaucoma, ischemia, and diabetic retinopathy. In recent decades, several pathways have been identified that modify the course of this selective neuronal cell death. The exact mechanisms remain unclear, but excitotoxicity seems to be important. Anti-excitotoxic drugs are neuroprotective in animal models of ocular hypertension and ischemia [144].

Antioxidant enzymes protect cells from ROS. In vitro and in vivo studies have shown that antioxidant enzyme mimetics, such as nitroxides, can improve defense against oxidative stress. Tempol is a nitroxide that easily penetrates cell membranes and also easily penetrates the blood–brain barrier [32]. It has been shown in vitro and in vivo to protect neuronal cells in neurodegenerative diseases; in models of brain injury, ischemic stroke, and Parkinson’s disease [145]. These diseases are very similar to retinal diseases such as glaucoma. In an in vitro model of retinal ganglion cell (RGC) damage by TNF-α and hypoxia, Tempol was shown to significantly increase RGC survival after mitochondrial damage [146]. TempolH showed similar neuroprotective effects in light damage models [147]. However, Tempol proved effective only in relatively high doses (≥100 mg/kg body weight) in vivo, which was unfavorable for animals [148]. Such high doses in conscious animals cause convulsions, hypotension, and agitation [80]. Greater effectiveness of nitroxide can be achieved by modifying the basic molecule, which allows it to be directed to a specific place in the cell or tissue. This condition is met by XJB-5-131 targeted to mitochondria. However, mitochondria are not the only place where ROS can be generated. Another place is the cell membrane, where plasma membrane oxidoreductase (PMORw) occurs. This enzyme is also found in neuronal tissue [149]. The lipophilic acyl ester of Tempol is neuroprotective in RGCs at lower doses than Tempol in the partial optic nerve crush model in rats (PONC) [150]. In another study, the acyl ester of Tempol was shown to be a potent neuroprotective agent in NMDA-induced RGC damage in rats. Interestingly, it was effective at a low concentration, at which Tempol was ineffective. However, these studies were conducted on a group of sexually immature rats. Therefore, an open question remains: what effect it will have in older rats [151]. In turn, Tempo reduced arrhythmia after experimentally induced cardiac ischemia in rats [152]. Nitroxide also had a beneficial effect in gerbils in which cerebral ischemia was induced by bilateral occlusion of the common carotid arteries [153]. Ischemic damage was identified postmortem using immunohistochemical (IHC) staining for nitrotyrosine, which indicates overproduction of peroxynitrite. In addition, polyADP-ribosynthetase (PARS) was determined as an indicator of DNA breaks. Intraperitoneal administration of Tempol before and after reperfusion led to a significant reduction in lipid peroxidation in the brain. In a subsequent study, the middle cerebral artery of rats was occluded and Tempol was administered within the first 20 min of reperfusion. Tempol administration led to a dose-dependent reduction in infarct size compared with the control group. Thus, Tempol provided significant neuroprotection after reperfusion in the rat model of transient focal ischemia [154].

In another model, intravenous administration of Tempol at the time of reperfusion significantly reduced the production of 2,3-DHBA (an indicator of hydroxyl radical production), reduced the level of cerebral TBARS, and reduced the size of the cerebral infarct. However, Tempol administered 15 min before reperfusion did not reduce either TBARS or the size of the infarct. These results indicate that Tempol administration at the time of reperfusion reduced lipid peroxidation by scavenging free radicals, which resulted in a reduction (more than 50%) of the infarct size [155]. Acute subdural hematoma (ASDH) is accompanied by increased production of ROS. Therefore, their elimination may be therapeutically beneficial. The use of Tempol significantly reduced the infarct volume compared to the control group (reduction of 42%). Nitroxide has shown significant neuroprotective effects in the rat ASDH model, which was associated with inhibition of ROS production [156].

Shock is a sudden, clinical life-threatening condition caused by a disproportion between the demand and supply of adequate oxygen (hypoxia), which results in the formation of toxic metabolites. During hypoxia, there is an overproduction of ROS, which damages cells and tissues. Septic shock was induced in rats using lipopolysaccharide. Intravenous administration of Tempol did not prevent circulatory collapse but prevented the development of renal and hepatic dysfunction [157].

### 3.11. Spin Immunoassay

In 1970, a radioimmunoassay (RIA) for morphine was published. In turn, the “heroin crisis” during the Vietnam conflict was an impetus for the development of a faster and safer version of RIA, spin label immunoassay, or spin immunoassay (SIA). Interestingly, the SIA opiate test was widely and effectively used, as can be concluded from 215,000 SIA results conducted on military personnel [158].

**Figure 11:**
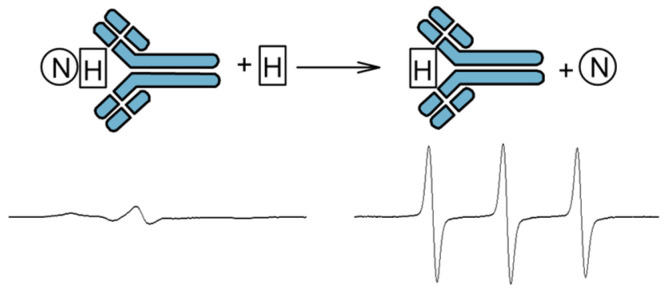
Principle of spin immunoassay. H-hapten, N-nitroxyl, antibody. On the left, is the EPR spectrum of the strongly immobilized marker, on the right, is the spectrum of the marker during free rotation.

The basis for the development of the spin immunoassay technique was the demonstration that spin-labeled haptens can bind to the appropriate antibody [159]. The authors showed that spin-labeled 2,4-dinitrophenol hapten (SL-DNP) binds with high affinity to antibodies against DNP. After introducing the anti-DNP antibody, a distinct change in the spectrum occurs, caused by the change in the rotational motion of SL-DNP, which was strongly limited after interaction with the antibody (Figure 11).

**Figure 12:**
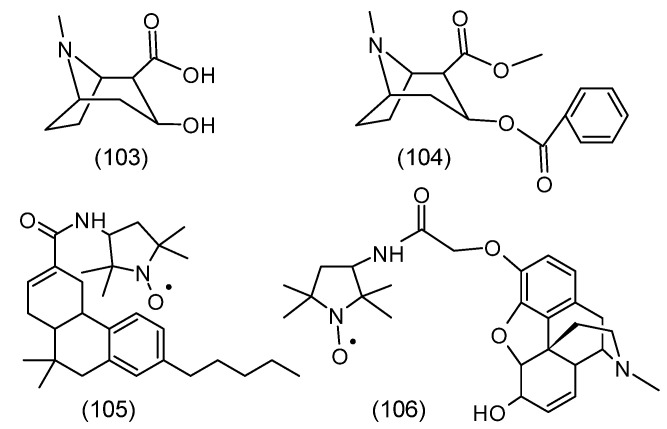
Ecgonine (**103**) and cocaine (**104**), and spin-labeled analogs of tetrahydrocannabinol (**105**) and morphine (**106**).

Thus, the hapten (morphine, heroin, ecgonine, and others) displaces the labeled drug from the complex (antibody-spin-labeled drug) (Figure 12). The diffuse EPR signal changes the EPR spectrum to a simple triplet of equal intensity. This technique has been used in the determination of drugs in saliva and urine. The use of this method requires spin-labeled drugs, such as morphine, heroin, cocaine, ecgonine, and others (Figure 12). Importantly, this technique does not require the separation of the compound bound and unbound to the antibody. The sensitivity of the method is 10^−6^ M, and the sample volume is 20 µL [160,161].

The method also allows the determination of drugs in plasma (progesteronediphenylhydantoine) and amphetamine, barbiturate, and methadone in urine [162,163].

### 3.12. Nitroxide in Cancer Cells and Tissues

Reactive oxygen species such as superoxide anion, nitric oxide, hydrogen peroxide, hypochlorous acid, peroxynitrite, and others are generated in normal cells. However, under hemostasis conditions, their concentrations are regulated by enzymatic (SOD, CAT, GPx, RG, peroxyredoxins, and others) and non-enzymatic (GSH, AA, a-TOH, b-Car, and others) systems. The main factors that determine their physiological or pathological effects are concentration and compartmentalization. In contrast to normal cells, cancer cells are characterized by overproduction of ROS. ROS are also important not only in the initiation of carcinogenesis but also in the subsequent stages of cancer development. ROS participate in the pathway in which a normal cell is transformed into a neoplastic cell and in tumor progression. ROS participates in HIF, NFκB, and sirtuin signaling as factors of cancer initiation and progression [164]. Nitroxides as antioxidants are not toxic to host cells and are toxic only to cancer cells. The introduction of an anticancer drug into the nitroxide molecule causes them to acquire new properties. Often, a nitroxide conjugate with an anticancer drug has several times stronger effects on cancer cells than the drug itself. In our previous work, we showed various spin-labeled anticancer drugs and their effects on examples of various cancers [165]. The use of nitroxides in cancer therapy is not only related to their cytotoxic effect on cancer cells but also to alleviating their side effects in other organs. Nitroxides can mitigate the effects of chemotherapeutic agents by limiting cardio-, hepato-, neuro-, and nephrotoxicity.

High ROS levels modify various signaling pathways that affect cellular metabolism. However, in cancer cells, high ROS levels are controlled by appropriate antioxidant defense mechanisms [166,167]. Increased ROS levels lead to reduced regulation of cellular antioxidant defense systems in tumor malignancy via various molecular targets, such as nuclear factor kappa-light-chain-enhancer of activated B cells (NF-κB) and nuclear respiratory factor 2 (Nrf2) [168,169]. High levels of ROS cause damage to vital macromolecules such as cellular DNA, proteins and lipids, leading to genomic instability and oncogenesis [170,171,172]. Excessive levels of ROS drive the cell toward apoptosis, but the high antioxidant capacity of cancer cells is enhanced to prevent the generation of high ROS concentrations and the maintenance of redox balance. Moreover, high antioxidant capacity stabilizes ROS production and activates pro-tumor signaling pathways without causing cell death in the tumor [166]. It is known that excessive ROS production can lead to various pathological conditions, such as neurological disorders, cardiovascular diseases, inflammation, autoimmunity, and cancer. ROS can initiate and support tumor development by promoting cancer cell proliferation and survival, facilitating pro-tumor signaling in the tumor microenvironment. As cancer cells become more resistant to ROS-releasing drugs, their increased antioxidant capacity reduces their susceptibility to treatment. However, excessive oxidative stress can lead to cancer cell death. Unlike normal cells, cancer cells have an altered redox environment that makes them more sensitive to changes in ROS or redox conditions [173]. Elimination of ROS by scavenging enzymes or increased ROS production may be effective options for cancer therapy [174].

In the case of many treatment-resistant tumors, combinations of doxorubicin (DOX) and docetaxel (DTX) are used. However, such chemotherapy causes side effects, e.g., cardiotoxicity, which results in oxidative damage to important macromolecules in the heart muscle. Chemotherapy performed in Sprague Dawley rats with DMBA (7,12-dimethylbenz(a)anthracene)-induced mammary tumors, using the DOX-DTX combination induced significant oxidative stress in the plasma. Dose-dependent oxidative damage to lipids and proteins and a decrease in thiols were observed. The drugs also increased the activity of SOD and NEAC. The introduction of 3-carbamoyl-2,2,5,5-tetramethylpyrroline-1-oxyl (Pyrroline) (**67**) to chemotherapy limited the oxidative stress generated by anticancer drugs. Although Pyrroline alone showed both antioxidant and pro-oxidant properties, in combination with drugs it protected macromolecules from oxidation [175]. Oxidative stress is important in carcinogenesis. The effect of Pyrroline and quercetin on breast cancer progression was studied. In Sprague Dawley rats, mammary tumors were generated with DMBA and then treated with intraperitoneal administration of Pyrroline or quercetin for 14 days. The histopathology of tumors, their size, and multiplicity were assessed. The effect of both antioxidants on heart tissue was assessed based on markers of oxidative stress and cleavage of poly(ADP-ribose) polymerase 1 (PARP 1). The median number of tumors and their volume at the end of the study were significantly smaller in both groups treated with antioxidants. Quercetin showed a better antioxidant effect in the heart, as it restored the GSH pool and reduced the level of hydroperoxides. However, both antioxidants did not prevent cardiomyocyte apoptosis. The attenuation of tumor progression by Pyrroline was comparable to the effect of quercetin. Importantly, no negative changes were observed in the hearts of animals after Pirolin application [176].

**Figure 13:**
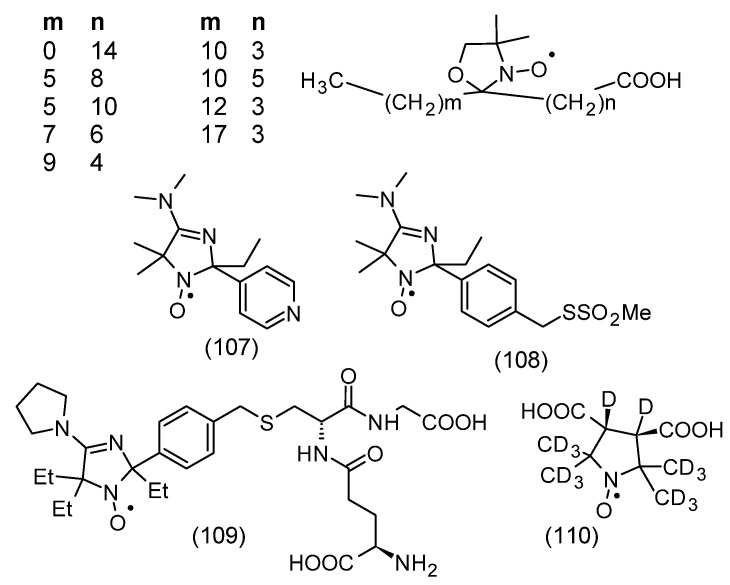
Different spin-labeled fatty acids. A paramagnetic group located at different carbon atoms in a hydrocarbon chain. Structure of probes and spin labels sensitive to the pH of the environment and spin labels: pH-sensitive nitroxides (**107**–**109**), deuterated nitroxide with a nitrogen nucleus 15N for EPR tomography oximetry (**110**) [179].

Anticancer drugs such as doxorubicin (DOX), paclitaxel (PTX), and docetaxel (DTX) used in the therapy of breast cancer induce side effects in other organs of the body. Although the blood–brain barrier (BBB) protects the brain from the effects of these drugs, its damage by oxidative stress results in the drugs being able to enter the brain. All the chemotherapeutic agents used induced OS, DNA damage, and changes in the expression of TNF-α, nNOS, and PARP-1 in the rat brain. Therefore, the inclusion of antioxidants in chemotherapy could potentially protect the brain from the toxicity of anticancer drugs. Although Pyrroline alone increased the activity of MnSOD and CAT and the expression of PARP-1, while decreasing the expression of TNF-α, it showed the best protective effects in combination with PTX. Piroline also partially attenuated the brain damage caused by DOX and taxanes [177]. In contrast to hydrophilic nitroxides, the biological properties of spin-labeled fatty acids/esters, such as doxyl stearate (DS): 5-DS, Met-12-DS, and 16-DS, used as spin probes in studies of natural and artificial membranes, have not been studied in detail so far (Figure 13). Studies have shown a cytotoxic effect of doxyl stearates on B14 cells compared to the natural endogenous antioxidant, α-tocopherol. Moreover, the effect of DS on cellular changes induced by the anticancer drug doxorubicin (DOX) has not been studied yet. Therefore, we investigated the cytotoxicity of DS and their ability to induce cell death and affect fluidity and lipid peroxidation (LPO) in the plasma membrane of immortalized B14 fibroblasts, used as model cancer cells susceptible to DOX-induced changes. The effect of DS on DOX toxicity was also studied and compared with the effect of the natural antioxidant, α-tocopherol. The highest cytotoxicity was shown by 5-DS, which was also reduced the fastest in cells. On the other hand, Met-12-DS was reduced the slowest. Nitroxides induced apoptosis and modified the fluidity of plasma membranes. On the other hand, the nitroxides used did not protect cells against cell death induced by DOX and even intensified DOX toxicity [178].

Nitroxides have also been used to treat existing tumors. Although Tempol is not toxic to normal cells, it has been shown to have antiproliferative effects on MCF-7 breast cancer cells [180,181,182]. Initially, cells accumulated in the G1 phase and then arrested in the G2/M phase, eventually undergoing apoptosis, as shown by DNA fragmentation studies. In a study conducted in HL60 leukemia cells, which do not produce p53 tumor suppressor, Tempol initiated a time- and dose-dependent increase in the expression of the downstream antiproliferative gene p21, resulting in arrest in G1 phase [183,184]. These results contributed to further studies related to the antitumor effect of Tempol on glioma cells in a mouse xenograft model in vitro and in vivo. It was shown that mice receiving Tempol showed increased signs of apoptosis, as confirmed by TUNEL analysis and direct visualization of cells, and additionally decreased vascularization in histological staining. The obtained results confirmed that the redox state of some tumors and their microenvironment was significantly changed concerning the state of normal tissue, which means that nitroxides can use this difference to destroy various malignant tumors [98].

### 3.13. Application of Nitroxides in Protein Research

Nitroxides in the spin labeling method allow the use of EPR techniques in the study of the structure and conformational dynamics of proteins during various types of interactions (e.g., enzyme substrate). Spin labels allow obtaining unique information about native proteins, such as the participation of hydrogen bonding, the effects of polarization of the microenvironment of protein microregions involved in the transfer process, determination of pH/pK, tracking of local electrostatic interactions of proteins using pH-sensitive labels, including measurements of the extracellular pH of the tumor in vivo (Figure 13) [185,186,187]. During various types of interactions, these factors can change and thus control the efficiency of biological processes. Examples include light-induced transfer of electrons and protons across photosynthetic membranes, as well as the formation of ion channels of bacterial toxins. The use of deuterated derivatives of nitroxides with a nitrogen nucleus 15N with EPR in a high field allows us to obtain information about the local polarity of the microenvironment [185]. Classical EPR spectroscopy using the spin labeling technique allows for determining changes in the structure of proteins after the action of various physicochemical factors (Figure 9). On the other hand, EPR spectroscopy in the form of pulsed double electron-electron resonance spectroscopy (PELDOR), is also known as double electron-electron resonance spectroscopy, DEER [188,189]. These methods, combined with directed spin labeling, allow for the study of the dynamics and visualization of conformational heterogeneity. It also allows modifying the distances between selected residues and determining the entire contact system, even for heterogeneous samples in an environment close to the physiological one. The PELDOR method allows us to obtain nanometer-scale distance distributions that provide information not only about the average conformation but also about the width of the native structure. Obtaining such information using classical structural biology techniques is often impossible [190].

One of the newer applications of the EPR method is structural studies inside living cells and visualization of biochemical processes in living tissues [191,192,193]. However, the presence of reducing agents such as ascorbate, reductases, NAD(P)H, and others leads to the reduction of nitroxides to hydroxylamines, which limits the use of classical nitroxides in biomedical research [106]. The introduction of sterically shielded nitroxides, more resistant to bioreduction compared to classical tetramethyl nitroxides, enables studies of living cells and tissues [100,111]. Many 2,2,5,5-tetraethylpyrroline-1-oxyl derivatives have also been synthesized as spin labels, which have been proposed for intracellular studies [194,195]. Interestingly, sterically shielded pyrroline-1-oxyl derivatives show lower resistance to reduction than similar pyrrolidine-1-oxyl derivatives [108,196].

EPR spectroscopy combined with spin labels enables studies of protein structural dynamics and protein–protein interactions in the cellular environment. Moreover, it enables direct observation of conformational changes in proteins inside cells [197]. Previously, such studies were difficult and sometimes even impossible due to the loss of the EPR signal associated with their intracellular reduction. Currently, the use of reduction-resistant labels enables studies of site-directed spin labeling (SDSL). The use of SDSL involves covalent attachment of a spin label to a specific position of a protein, DNA, RNA, and lipid macromolecule, and then studies using continuous-wave EPR (cw-EPR) or pulsed dipole EPR [198,199,200,201]. Cw-SDSL-EPR measurements are most often conducted at room temperature, which allows us to obtain information on conformational changes, solvent accessibility, and interactions between macromolecules [198,202]. In contrast, the pulsed EPR technique, mainly Double Electron-Electron Resonance (DEER), enables measurements of the distance between two labels, the same or different spin labels, in the range of 1.5–8 nm. This method provides information related to conformational transitions or association of biomolecules [203,204].

Since spin-label molecules are small, their presence causes less disruption to protein structure and function. For example, water accessibility to a spin-labeled side chain can be determined using continuous wave (cw) EPR in the X-band. The newly developed Overhauser dynamic nuclear polarization (ODNP) method is particularly useful. Furthermore, spin labels can be used orthogonally to other spin probes [195]. SDSL-EPR and DEER spectroscopies enable structural studies in cellular environments (intracellular EPR) [191]. In the last decade, new spin labels have been synthesized and new methods for intracellular studies, using EPR spectroscopy, have been developed. A variety of SDSL-based methods have shown that EPR spectroscopy can be useful for studying the conformation state and dynamics of proteins and nucleic acids in natural cellular environments [205,206]. The extended lifetime of M-TETPO (3-maleimidoethyl-2,2,5,5-tetraethylpyrrolidine-1-oxyl) (**96**) has enabled the study of the structural properties of the chaperone in the absence and presence of its binding partner, at endogenous concentrations, directly inside cells [194]. Compared to directed spin labeling methods, spin labeling of proteins by incorporating unnatural spin labels has several potential advantages [202]. Spin-labeled unnatural amino acids provide new abilities for spin-labeling proteins by suppressing nonsense codons, introducing stop codons, or genetically encoding spin-labeled unnatural amino acids in living cells [207]. However, the short lifetime of nitroxides is a serious limitation of this method. The use of new nitroxides, resistant to reduction will enable their application in the study of the structure and function of proteins expressed in biological environments [208].

A spin label attached to a protein or peptide changes its mobility (rotation), which is dependent on its immediate environment. Its total mobility is the sum of the label’s movements relative to the peptide backbone, changes in the conformation of the carbon backbone, and the rotational movement of the entire protein or peptide. The EPR spectrum of a spin label bound to a protein or peptide reflects its rotations. Spectrum analysis can be used to predict the local secondary structure of proteins and peptides [198,202,209]. In addition, changes in the spin label’s mobility can be taken into account in the study of peptide binding to the membrane [210]. The dynamics of the spin label’s protein link is dependent on the spatial properties of the molecule’s microenvironment, which allows for the study of changes in this microenvironment. They are the result of possible conformational changes, ligand binding, or differences in membrane lipid sharing or intracellular or extracellular environment in the case of transmembrane proteins [211]. One of the most common applications of SDSL-EPR is the determination of spin distances in doubly labeled systems by pulsed dipole spectroscopy (PD). The range of PD distance measurement by DEER in spin-labeled proteins in aqueous environment ranges from 1.7 nm to 6.0 nm in spectrometers operating in the X-band (9.5 GHz), while much better sensitivity of the method is achieved using the Q-band (35 GHz). Although improvements of the method and the use of protein deuteration extend the upper range of distances to 8 nm and potentially 10 nm [202]. In turn, the topology of a protein present in the membrane can be determined relative to the membrane, using microwave power saturation in spin labeling measurements directed locally in EPR spectroscopy [212,213]. This approach can also be used to identify functional domains in membrane proteins [214]. The SDSL method in EPR spectroscopy is used to study membrane proteins. It allows the study of the dynamics and topology of membrane proteins, the local secondary structure of membrane proteins and the measurement of SDSL distances of membrane proteins [198,202,210,211,215]. Membrane proteins play a key role in signal transduction, participate in the transport of substances across biological membranes and are important in various directions of cellular activity [216]. Membrane protein malfunction is associated with numerous defects, disorders, and diseases in humans [217,218]. Membrane proteins participate in interactions with the lipid bilayer, maintaining membrane integrity and its functional properties. Protein helices can be arranged parallel to the membrane surface and/or cross it at various angles and also form reentrant loops.

### 3.14. Site-Directed Spin Labeling Inside Cells

In the DEER method applied to whole cells, SDSL enables distance measurements that can provide information on the structural dynamics of proteins in their natural environment and their physiological function. In one study, previously spin-labeled macromolecules with a maleimide spin label were transfected into model cells, such as Xenopus oocytes [205,206]. However, these cells are an unnatural environment for proteins other than those of Xenopus origin, i.e., they are devoid of natural processing of the target protein by folding, transport, post-translational modifications, and degradation. DEER combined with SDSL was used to study proteins inside eukaryotic cells, in *Xenopus laevis* oocytes. Microinjection of the SDSL-ubiquitin derivative into oocytes allowed in situ analysis of interspin distances of proteins [219]. Labeling the protein with a spin label and introducing it into the cell using the electroporation method, the characteristics of the cytoplasmic protein in its native environment in *Escherichia coli* (*E. coli*) cells were determined [197]. It was shown that the introduced protein can activate its partner in the cell. The local and global dynamics of the protein were also determined using the EPR method. By labeling different regions of the protein with maleimido-nitroxide-Proxyl and simulating CW-EPR spectra in the cell, the influence of the environment inside the cell on the local dynamics of the protein was quantitatively estimated. A site-specific effect on the mobility of the protein in the cytosol was demonstrated. Similar studies were carried out in the extracellular environment in the presence of crowding factors [197]. In other studies, the bacterial outer membrane protein was spin-labeled with MTSL (1-oxyl-2,2,5,5-tetramethylpyrroline-3-methyl)methanethiosulfonate, which is most often used in SDSL studies due to its thiol specificity. MTSL was bound to cysteine residues of the cobalamin transporter. Pulse-wave EPR spectroscopy was used to measure the interspin distances to the spin-labeled cobalamin. Conformational changes in the proteins after ligand binding were determined. Since the studies were conducted on the cell surface, there was no problem with the reduction of nitroxide, which is observed in the cytoplasm. In turn, the labeled amino acid 9 (SLK-1) was used for the biosynthesis of thioredoxin in *E. coli* in the cytoplasm of living cells by translation with the expanded genetic code [211]. Studies of the conformational changes or protein-protein/ligand interactions at the surface-exposed sites of membrane protein complexes in whole cells and native membranes in their native environment have been shown. The analysis performed showed similar interspin distances between whole cells, outer membranes and synthetic vesicles [220]. The same research group developed a new approach for high-resolution distance measurements in the nanometer range to study the structure and conformational dynamics of membrane proteins. Outer membrane proteins in intact *E. coli* membranes and native membranes were used. Spin labeling was performed using the MTSL and interspin distances were determined in situ using double electron-electron resonance spectroscopy (PELDOR or DEER). This method has enabled the observation of protein-ligand/substrate interactions, oligomerization, and conformational dynamics of outer membrane proteins in the native outer membrane and intact *E. coli* [221].

Site-directed spin labeling in EPR spectroscopy is particularly well suited to the study of membrane proteins. Conformational states that arise interchangeably are recorded as distinct populations in the EPR spectrum, allowing the identification of protein regions that are in dynamic exchange. Protein dynamics and conformational exchange determine many important activities, such as protein-protein and protein-ligand interactions, enzymatic activity, and protein allostery [222]. Although protein crystallography enables high-resolution studies of proteins, for a large number of membrane proteins, these models do not always reflect the actual structures due to conformational exchange, especially when the protein is in its native bilayer environment. Studies of the *E. coli* cobalamin (vitamin B12) transporter have revealed that BtuB, the energy-coupling segment of this protein, changes conformation, which is substrate-dependent, to associate this outer membrane protein with the inner membrane protein TonB. Using EPR spectroscopy, it was revealed that the energy-coupling segment is in equilibrium between ordered and disordered states. In contrast, substrate binding causes a shift in the equilibrium state toward the unfolded state. However, neither the equilibrium state nor the changes induced by substrate binding are observed in the crystal structures of BtuB. Using the SDSL method, it was found that the conformational change also occurs in other regions of BtuB, as well as in other proteins of this transporter family [222].

The conformational exchange was also studied in the case of the plasma membrane SNARE protein, syntaxin 1A, in which the transitions are controlled by regulatory proteins, munc18. The regulation of the transitions on a time scale ranges from microseconds to milliseconds. The main purpose of the neuronal SNARE is likely the assembly of SNARE and the release of neurotransmitters [222]. The genetic coding of Proxyl spin-labeled amino acids in *E. coli* is presented. Their introduction into the cell enables the biosynthesis of spin-labeled proteins without any chemical labeling. Such amino acids can be introduced in many specific places of the protein and are stable in cells for a longer time. This enables measurements of intramolecular distances in proteins using the DEER method. Moreover, the signal of the spin-labeled protein can be selectively detected in cells. This method shows new perspectives for the study of intracellular proteins using the EPR method [207].

### 3.15. Application of Nitroxides in EPR/MRI

The resulting nitroxide, Teepone (2,2,6,6-tetraethyl-4-oxopiperidine-1-oxyl) (**68**) was also useful for in vivo three-dimensional EPR and MRI of the mouse head. Its half-life was more than 80 min, which was considerably longer compared to its counterpart containing methyl groups at the 2 and 6 positions (half-life of several minutes). Interestingly, Teepone (**68**) was rapidly transformed in the presence of rat liver microsomal fractions and NADPH to a diamagnetic compound. Further studies showed that the rapid loss of Teepone paramagnetism was not related to its reduction to hydroxylamine but to the oxidation of the methylene group in the alpha position to carbonyl by the perferryl species P450-Fe^5+^=O. Furthermore, the abstraction of the hydrogen atom by P450 on ethyl substituents, which can also lead to dehydrogenation or hydroxylation products, leaves an active nitroxyl group but affects the EPR signal line width [114].

Reactive oxygen species (ROS) are produced by living organisms during normal cellular metabolism. However, their level is regulated by enzymatic and non-enzymatic systems under normal physiological conditions. However, in pathology, excessive ROS production and/or reduced antioxidant defense led to cardiovascular (heart attack, atherosclerosis) and brain (stroke, Parkinson’s disease, and Alzheimer’s disease) disorders. In the case of the brain, noninvasive assessment of the redox state is necessary to monitor the disease state and oxidative damage. Continuous electron paramagnetic resonance imaging (CW-EPR) using nitroxides, sensitive to redox stress, is a reliable technique for visualizing the redox state changes by oxidative stress in vivo. The introduction of new EPR imaging systems, taking into account site-specific distribution and probe kinetics, can provide more detailed information compared to previous EPR imaging systems. This method has proven to be particularly useful in studies for imaging nitroxide in rodent brains. In this method, classical nitroxides such as Tempol, CTPO, 3-carbamoyl-Proxyl (CMP) (**65**), 3-carboxy-Proxyl (COP) (**64**), 3-hydroxymethyl-Proxyl (HMP) similar to nitroxide (**61**), and 3-methoxycarbonyl-Proxyl (MCP) (**111**) were used [223].

**Figure 14:**
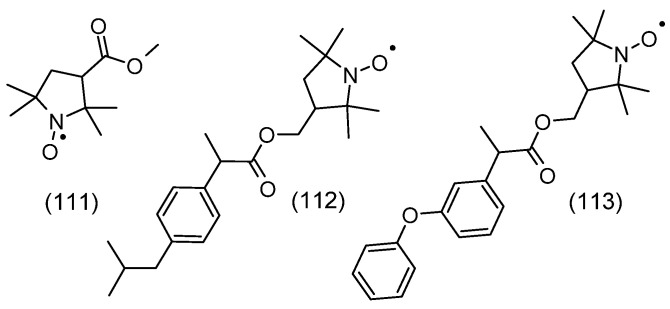
Nitroxides crossing the blood–brain barrier (BBB). 3-carboxy-Proxyl methyl ester (**111**) and theranostic probes: spin-labeled ibuprofen (**112**) and spin-labeled ketoprofen (**113**).

Free radical reactions in the brain are an important issue in recent medical research. Nitroxides are important for imaging the redox state of the brain using the EPR technique, as they cross the blood–brain barrier (BBB), are retained in the brain, and are sensitive to the redox state under oxidative stress conditions. These conditions are met by the above-mentioned probes except for COP. Interestingly, EPR images obtained using MCP and HMO probes showed regional differences in the rate of decay in the rat brain. EPR images showed that MCP and HMO are mainly distributed inside the brain, while COP is mainly distributed outside the brain (Figure 14) [223]. Theranostic probes combine therapeutic and diagnostic imaging capabilities in one molecule and may be interesting for magnetic resonance imaging (MRI) studies. Such compounds have, on the one hand, nitroxide-based contrast agent properties, and on the other hand, nonsteroidal anti-inflammatory effects. Theranostic probes were created by synthesizing ibuprofen or ketoprofen with a 3-hydroxymethyl-2,2,5,5-tetramethylprolidine-1-oxyl hydroxyl group (Figure 14). MRI studies of mouse heads showed that the probes cross the blood–brain barrier (BBB), which results in contrast enhancement in the mouse brain. This process was maintained for quite a long time (half-life of about 40 min). This time is longer than the time represented by most nitroxyl pyrrolidine derivatives. The therapeutic potential of both theranostic probes was studied in the lipopolysaccharide (LPS)-induced encephalitis model. The production of nitric oxide as a marker of inflammation in the brain of mice with sepsis induced by LPS was significantly reduced after administration of either probe, which is important evidence that they also have anti-inflammatory effects. Thus, the obtained theranostic probes act as redox-sensitive contrast agents and as anti-inflammatory drugs in the brains of mice with sepsis [224].

Nitroxides used as MRI contrast agents have important advantages compared to transition metal ion-based contrast agents. These include low toxicity, chemical modification possibilities, and easy elimination from the body [225]. Their disadvantage is that they have only one unpaired electron, while, for example, the gadolinium (III) ion has as many as 7 unpaired electrons, which contributes to a much lower relaxivity compared to nitroxides [226]. In addition, nitroxides are reducible under physiological conditions, which is another disadvantage in their use as contrast agents for magnetic resonance imaging [7]. However, the properties of nitroxides have recently been greatly improved. On the one hand, the introduction of ethyl groups in place of methyl groups in the vicinity of the nitroxyl group has limited their reducing properties. On the other hand, they were incorporated into polymeric scaffolds, which allowed the creation of new paramagnetic contrast agents with high relaxivity. In addition, the polymeric scaffold can prevent the reduction of radicals in vivo, giving a shielding effect [227]. Nitroxides were encapsulated or anchored on various, bulky scaffolds, such as chitosan, cyclodextrin, albumin, carbon nanotubes, polymers, and dendrimers [227].

Dendrimers belong to a class of special polymers that form a monodisperse and well-characterized macromolecular structure, usually forming a spherical shape due to their symmetric and regular structure. Dendrimers usually have different functional groups at the ends of the branches and create internal cavities between the branches [228]. The molecular structure makes dendrimers a good scaffold for the preparation of nitroxide-based contrast agents with extraordinary physical and chemical properties. Nitroxides can be anchored on the dendrimer surface using various chemical modifications to obtain higher molecular relaxivity. This is achieved by multiplying the number of bound nitroxides. In addition, the dendrimer creates a protective shield effect that limits the availability of endogenous reducing agents. The precise number of functional groups and the controlled size of the molecule make dendrimers suitable macromolecules for scaffold anchoring of nitroxides to create excellent contrast agents. By synthesizing successive generations of dendrimers from generation to generation 4 based on PPH, it was possible to obtain, respectively, 6, 12, 24, 48, and 96 Tempo units on the periphery [229]. Moreover, nitroxides combined with higher-generation dendrimers with 8–32 radicals showed high resistance to a reduction in biological systems [230,231]. It has been shown that the linker between the dendrimer and the nitroxide is crucial in modulating the interactions between the radicals. This opens new possibilities in tailoring the properties of dendrimers as contrast agents [231]. Although most of them are based on PPH dendrimers, polyurethane dendrimers, oligoethylene glycol dendrimers, and oligo(styryl)benzene dendrimers have also been obtained [232,233,234], which have become new contrast agents in MRI. Radical dendrimers containing nitroxides, especially Proxyl, showed a significantly lower reduction rate and also higher stability compared to the free radical. Radical dendrimers with high relaxivity and water solubility are excellent contrast agents in MRI. Moreover, control over the structure of radical dendrimers, i.e., appropriate generation and size, creates great possibilities in their biodistribution in vivo.

Dendrimers have many applications in biomedicine, which is related to their spatial structure. The introduction of nitroxides into their structure allowed obtaining contrast agents with high relaxivity, which can replace traditional metal-based ones. Dendrimers used in imaging diagnostics reduce the dose required to obtain images compared to contrast agents containing metals. It was shown that one molecule of the G3-Tyr-PROXYL radical dendrimer had a 4-fold higher relaxivity value than Gd-DTPA. Ex vivo MRI studies conducted on mice showed very good relative contrast enhancement after local injection of the G3-Tyr-PROXYL dendrimer compared to Gd-DTPA. Interestingly, four generations of radical dendrimers were characterized by very low cytotoxicity. These studies have shown that such radical dendrimers can be used as MRI contrast agents for biomedical applications. Moreover, the control of the structure of these dendrimers creates new possibilities for modulating their biodistribution profiles [231]. A G5 dendrimer based on a cyclotriphosphazene core and lysine-derived branched units was synthesized. This dendrimer contains 192 units of Tempo nitroxide. The Vero cell viability study revealed that G3 and G3.5 showed good biocompatibility. In vivo MRI measurements performed in mice revealed renal excretion of G3.5 and selective accumulation in glioblastoma multiforme tumors [235]. Nanometric dendrimers are characterized by unique interactions with cell membranes. These interactions can be monitored in situ by spin labeling. Two nitroxides 4-(N,N-dimethyl-N-dodecyl)ammonium-2,2,6,6-tetramethylpiperidine-1-oxybromide (CAT12), and Tempo were covalently attached to newly synthesized heterofunctional dendrimers to provide information on dendrimer–membrane interactions. Computer-assisted EPR analysis showed good agreement between the results obtained for spin probe and spin-label experiments. Both polar and hydrophobic interactions were demonstrated. In contrast, in the case of the dendrimer–lecithin liposome system, more polar interactions between surface groups were observed. Minor changes in the structure of dendrimers greatly modify their ability to interact, which can also be translated into their anticancer activity [236]. Polyurethane dendrimers containing Tempo, which have antioxidant properties, also showed anticancer properties. The G4 generation of these dendrimers showed significant anticancer activity and protective effect against oxidative cell damage [232,233].

The third generation of water-soluble poly(phosphorohydrazone) (G3-Tyr-Proxyl) radical dendrimers containing 48 Proxyl units were studied ex vivo and in vivo in brain tumors (in immunocompetent, orthotopic glioblastoma multiforme of GL261 (GB) mice). This dendrimer provided adequate contrast enhancement in GL261 GB mouse tumors at a dose of 4-fold lower, similar to commercial Gd-containing contrast agents (standard dose 0.1 mmol/kg). In the case of the dendrimer, no symptoms of toxicity were detected in vivo. Interestingly, the dendrimer showed selective accumulation in brain tumor tissues, which allowed imaging in more than 2.5 h in contrast to Gd chelates. In addition, the dendrimer showed high radical stability in biological media, in the order of hours instead of minutes, which is characteristic of isolated radicals. Therefore, the G3-Tyr-PROXYL radical dendrimer may be an important contrast agent in MRI analysis of GB in vivo [237]. Biocompatible dendrimers based on oligoethylene glycol containing 5 and 20 nitroxides (OEG Gn-Proxyl radical dendrimers (*n* = 0, 1)) were obtained. Importantly, these dendrimers were water-soluble, such a feature is often a problem, especially in the case of large-generation dendrimers. These properties make them good contrast agents for MRI applications, such as infectious disease diagnosis and monitoring [234]. Tempo-containing G4 dendrimer was shown to have significant antitumor activity in A549 lung adenocarcinoma cells. In contrast, it did not induce cell death in L132 normal lung cells. Furthermore, this dendrimer showed antioxidant and cytoprotective effects compared to N-acetylcysteine [232].

Recently, dual imaging probes have been described to improve the sensitivity and accuracy of diagnostic imaging like MRI and optical fluorescence imaging (OFI). Dendrimers used oligo(styryl)benzene (OSB) cores, which exhibit their fluorescence, and Tempo nitroxide bound to their surfaces, six radical dendrimers were synthesized this way. It was shown that these new ones are good contrast agents in MRI in vitro, and on the other hand, they also exhibit fluorescence [238]. Anticancer studies of dendrimers, such as poly(amidoamine) and poly(propyleneimine) with doxorubicin, paclitaxel, imatinib, sunitinib, cisplatin, melphalan, and methotrexate were also carried out showing their higher efficiency compared to drug molecules. In turn, anti-inflammatory therapy was associated with dendrimers containing ibuprofen, indomethacin, piroxicam, ketoprofen, and diflunisal. In the case of antibiotic-resistant bacterial strains, dendrimers filled with fluoroquinolones, macrolides, beta-lactamines, and aminoglycosides were used, and preliminary studies showed promising effects. For antiviral therapy, combinations of dendrimers with tenofovir, maraviroc, zidovudine, oseltamivir, and acyclovir were used. In addition, conjugates of dendrimers with drugs were also used in cardiovascular therapy [239].

**Figure 15:**
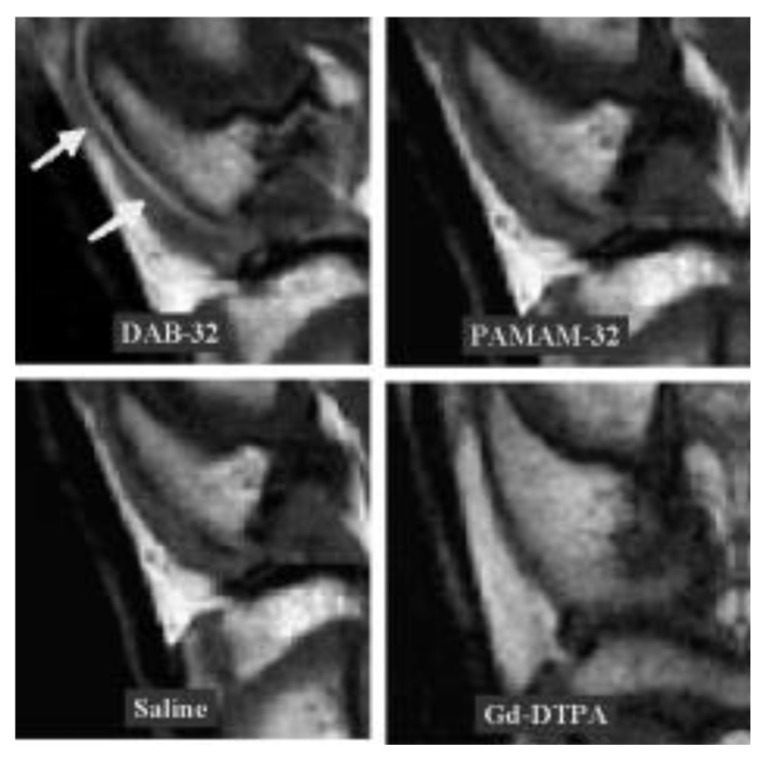
Sagittal T1-weighted images of stifle joints obtained 3 h after intra-articular injection of the agents. Arrows designate cartilage enhancement with DAB-32. Joint with PAMAM-32 unenhanced [240]. (Posted with the author’s permission).

Contrast agents are of great importance in the assessment of physiological and pathological changes in various disease states. Unfortunately, currently used gadolinium chelates are highly toxic. Great hopes are associated with the use of appropriate polymers, biopolymers, including proteins and peptides, which can be used as components of adaptive nanometric formulations that can be loaded with nitroxides. Although nitroxides are reduced in vivo, contrast agents have been used in MRI spectroscopy. To extend their lifetime, high doses were used, which were not neutral to organisms but compensated for their relatively poor relaxivity compared to Gd chelates (e.g., Gd complex with diethylenetriaminepentaacetic acid, Gd-DTPA). This problem was solved by using complexes of dendrimers with nitroxides. Succinimide ester-Proxyl (**87**) was used to introduce nitroxide residues into the DAB dendrimer (polypropylenimide dendrimer of the fourth generation). 3-methylamino-Proxyl, a compound similar to nitroxide (**34**) in the form of a quaternary ammonium salt [230], was used to introduce nitroxide residues into the PAMAM dendrimer (polyamidoamine of the third generation). The uptake of the dendrimer–nitroxide complex by rabbit knee joint cartilage was assessed using in vivo MRI spectroscopy. The injection of the dendrimer–nitroxide complex showed in MRI a high affinity of the DAB dendrimer–nitroxide complex for normal rabbit joint cartilage compared to the Gd-DTPA complex and the dendrimer–nitroxide (PAMAM) complex. Moreover, it was shown that nitroxides combined with DAB and PAMAM had higher relaxivity than the Gd-DTPA complex. Both dendrimer complexes contained a maximum of 32 Proxyl nitroxide residues. Higher numbers of nitroxides per dendrimer increased relaxivity compared to a single nitroxide, which allows for differential contrast at a reduced dose in MRI assessments [230]. After injection of the dendrimer–nitroxide complex, the synovial fluid was bright at the beginning of imaging with a gradual signal decay over 3.3 h (Figure 15).

Enhancement of the articular cartilage was visible as a thin bright line along the articular surfaces of knee joints previously injected with DAB dendrimer–nitroxide complexes. Interestingly, no cartilage enhancement was observed in knee joints after injection of the PAMAM dendrimer–nitroxide complex, nor Gd-DTPA, nor saline [230,240].

The study conducted by Winalski and colleagues demonstrated that all dendrimer–nitroxide complexes, except for those containing the highest number of bound radicals linked to the dendrimer, significantly strengthened articular cartilage [241]. This effect was evaluated using the MRI method. This effect was assessed using MRI. Dendrimers containing the highest number of nitroxide residues did not adhere to articular cartilage. Interestingly, the half-lives of the complexes were long enough to allow in vivo MR imaging. The relaxation measurements performed per dose were 3.5 to 68 times greater for the dendrimer–nitroxide complexes than for the Gd-DTPA complex. Histological examination of the joints showed minimal inflammation and necrosis of the synovial membrane 24 h after the injection. These symptoms were similar for all dendrimer–nitroxide complexes, including the Gd-DTPA complex. This method could be used in the assessment of osteoarthritis, which is characterized by degeneration and loss of articular cartilage. The initial stage of the development of the disease process is associated with the loss of glycosaminoglycan (GAG) molecules in articular cartilage, which progresses with the severity of the disease. Changes in the structure of articular cartilage are associated with the loss of its biomechanical properties. Since changes in cartilage appear in the early stages of the disease, it is of great importance to use a noninvasive method that will allow the detection of these changes, as well as monitor the progress in the development of cartilage matrix abnormalities [242]. Such a method may be MRI combined with contrast agents based on nitroxides, especially spatially shielded. Recently, using a dendrimer–Fe_3_O_4_–nitroxide complex in MRI spectroscopy, it was shown that the autophagy status of tumors could be effectively monitored in vivo [243]. Regulation of autophagy level is essential for improving chemotherapy efficacy. ROS generated by autophagy in tumor cells leads to the reduction of nitroxides, thus weakening the T1 signal. On the other hand, Fe_3_O_4_ molecules, which are not sensitive to ROS, give a stable T2 signal. To avoid T1 signal quenching, NAC was added, which rapidly inactivated ROS. By comparing the ratio of T1 to T2 intensity in the dendrimer–Fe_3_O_4_–nitroxide complex, the autophagy status in vivo within tumors can be estimated in real time. The complex was dosed with doxorubicin (Dox) together with the autophagy inhibitor, 3-methyladenine (3-MA), which led to high antitumor activity of the DOX complex in cells and mice with tumors. In tumor tissues of mice treated with the dendrimer–Fe_3_O_4_–nitroxide–DOX-3-MA complex, most tumor cells shrank and died. It was also shown that the dendrimer–Fe_3_O_4_–nitroxide–DOX-3-MA complex was characterized by the best therapeutic, antitumor effect. After 30 days of treatment, the pathological features of each organ were determined in the collected major organs of mice. The histological structures of the major organs (heart, liver, spleen, lungs, and kidneys) did not differ significantly from those in healthy mice, which indicates that the applied treatment did not cause significant damage to the major organs of mice [243].

In order to improve the sensitivity of MRI, the aim of which is to distinguish the examined tissue from the background, more and more new methods are being introduced. One of them is Overhauser dynamic nuclear polarization (ODNP)-enhanced MRI (OMRI). In addition to the use of dendrimers as MRI contrast agents, other polymers and biomolecules are also in use. One of them is human serum albumin (HSA), to which 21 nitroxide residues have been introduced [244]. It was shown that such a nitroxide-loaded molecule (HSA–nitroxide) showed similar toxicity to native HAS, also nitroxide residues bound to albumin were less susceptible to reducing agents (ascorbic acid). This applies to nitroxides containing methyl groups in positions 2,2,5,5 of the pyrrolidine ring, as well as those containing ethyl groups. The lowest reduction rate constant (k = 0.0007 M^−1^s^−1^) was shown by the conjugate containing 3-maleimidoethyl-2,2,5,5-tetraethylpyrrolidine-1-oxyl, which is why the HSA–nitroxide conjugate is highly stable in vivo. This constant was 2.5 times lower than that obtained for the conjugate of the radical dendrimer with four similar nitroxide molecules. The nitroxide-loaded albumin molecule is a radical hyperpolarizing contrast agent for OMRI. Phantoms were used to demonstrate the potential application of HSA–nitroxide and to determine the effectiveness of 1H Overhauser dynamic nuclear polarization (ODNP) in liquids at ultralow magnetic field for HSA–nitroxide conjugates. Interestingly, HSA–nitroxide molecules did not show ODNP enhancement. However, under proteolytic conditions, that simulated cancer tissue, HSA–nitroxide molecules fragmented to form lower molecular weight proteins capable of activating ODNP and leading to a 40–50% amplification of the spectroscopic signal. Biodegradation of the HSA–nitroxide complex resulted in the release of more nitroxide molecules, which resulted in a significant enhancement of the Overhauser effect [244].

## 4. Conclusions and Perspectives

**Figure 16:**
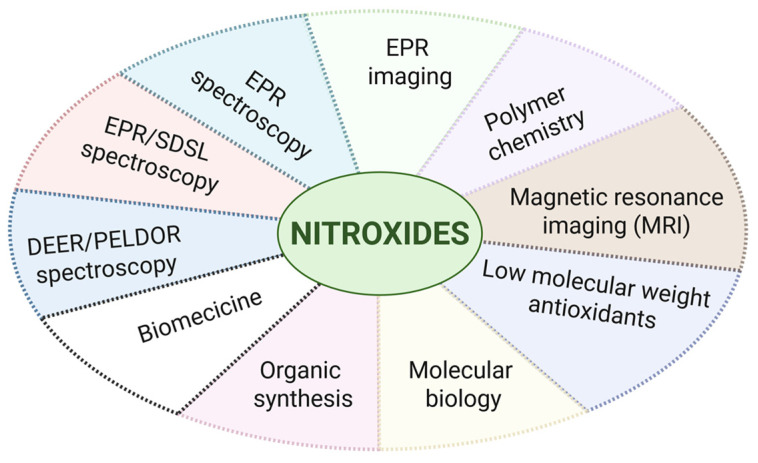
Applications of nitroxides.

Currently, many new spin labels and probes are available, which enable the selection of new strategies in protein studies using the SDSL EPR method. The synthesis of new spatially shielded nitroxides enables the further development of these studies, especially in living cells in their natural biological environment. This is one of the most important perspectives for the development of the SDSL EPR method in EPR spectroscopy in cells (Figure 16). On the other hand, the requirements for introducing spin-labeled proteins into cells do not fully meet expectations, because they are not endogenous proteins naturally processed in the cells. However, the perspective of using genetically encoded spin-labeled amino acids offers progress in the study of proteins in the cytoplasm, i.e., in their natural environment. These new approaches contribute to the development of new structural studies in cell biology using EPR spectroscopy and provide new information on the physiological functions of proteins. The development of new generations of nitroxide-based radical dendrimers opens new perspectives for their applications as highly relaxant contrast agents in MRI spectroscopy (Figure 16). Particularly interesting and promising will be the introduction of spatially shielded nitroxides into new nitroxide dendrimers.

Many experiments conducted using classic nitroxides will likely be repeated using sterically protected nitroxides. Importantly, a large number of experiments were carried out in a relatively short time due to the reduction of nitroxides; for the same reason, many other studies could not be carried out at all. The use of a new generation of nitroxides using polymers (dendrimers) creates new possibilities of application in molecular biology/biomedicine research, but also in spectroscopic techniques using EPR and MRI spectroscopy. An example here is the monitoring of autophagy of tumor cells in real time with simultaneous delivery of chemotherapeutic drugs. This method can be an innovative and effective strategy for the treatment of tumors with potential prospects for clinical application. Another example is the use of dendrimer–nitroxide complexes in the imaging of the knee and other joints, as well as the possibility of targeted drug delivery by dendrimer–nitroxide complexes.

## Data Availability

Not applicable.
